# Developmental programming of DNA methylation and gene expression patterns is associated with extreme cardiovascular tolerance to anoxia in the common snapping turtle

**DOI:** 10.1186/s13072-021-00414-7

**Published:** 2021-09-06

**Authors:** Ilan Ruhr, Jacob Bierstedt, Turk Rhen, Debojyoti Das, Sunil Kumar Singh, Soleille Miller, Dane A. Crossley, Gina L. J. Galli

**Affiliations:** 1 Division of Cardiovascular Sciences, School of Medical Sciences, University of Manchester, Manchester, M13 9NT UK; 2 Department of Biology, University of North Dakota, Grand Forks, ND 58202 USA; 3 Department of Biological Sciences, University of North Texas, Denton, TX 76203 USA

## Abstract

**Background:**

Environmental fluctuation during embryonic and fetal development can permanently alter an organism’s morphology, physiology, and behaviour. This phenomenon, known as developmental plasticity, is particularly relevant to reptiles that develop in subterranean nests with variable oxygen tensions. Previous work has shown hypoxia permanently alters the cardiovascular system of snapping turtles and may improve cardiac anoxia tolerance later in life. The mechanisms driving this process are unknown but may involve epigenetic regulation of gene expression via DNA methylation. To test this hypothesis, we assessed in situ cardiac performance during 2 h of acute anoxia in juvenile turtles previously exposed to normoxia (21% oxygen) or hypoxia (10% oxygen) during embryogenesis. Next, we analysed DNA methylation and gene expression patterns in turtles from the same cohorts using whole genome bisulfite sequencing, which represents the first high-resolution investigation of DNA methylation patterns in any reptilian species.

**Results:**

Genome-wide correlations between CpG and CpG island methylation and gene expression patterns in the snapping turtle were consistent with patterns observed in mammals. As hypothesized, developmental hypoxia increased juvenile turtle cardiac anoxia tolerance and programmed DNA methylation and gene expression patterns. Programmed differences in expression of genes such as *SCN5A* may account for differences in heart rate, while genes such as *TNNT2* and *TPM3* may underlie differences in calcium sensitivity and contractility of cardiomyocytes and cardiac inotropy. Finally, we identified putative transcription factor-binding sites in promoters and in differentially methylated CpG islands that suggest a model linking programming of DNA methylation during embryogenesis to differential gene expression and cardiovascular physiology later in life. Binding sites for hypoxia inducible factors (HIF1A, ARNT, and EPAS1) and key transcription factors activated by MAPK and BMP signaling (RREB1 and SMAD4) are implicated.

**Conclusions:**

Our data strongly suggests that DNA methylation plays a conserved role in the regulation of gene expression in reptiles. We also show that embryonic hypoxia programs DNA methylation and gene expression patterns and that these changes are associated with enhanced cardiac anoxia tolerance later in life. Programming of cardiac anoxia tolerance has major ecological implications for snapping turtles, because these animals regularly exploit anoxic environments throughout their lifespan.

**Supplementary Information:**

The online version contains supplementary material available at 10.1186/s13072-021-00414-7.

## Introduction

The environment that an organism experiences in early life can have profound and long-lasting effects on their phenotype. This phenomenon, termed developmental plasticity, allows animals to permanently alter their morphology, physiology and behaviour in response to environmental signals [1]. In many cases, developmental plasticity provides organisms with a powerful mechanism to cope with environmental heterogeneity later in life [2]. However, unexpected or severe environmental stress during development can produce maladaptive phenotypes that increase disease susceptibility [3]. Despite the profound ecological implications of developmental plasticity, the underlying cellular and molecular mechanisms remain poorly defined.

Due to the profound health implications, most studies investigating developmental plasticity have focused on mammalian models of disease [4]. However, environmental variation during development is much more common in ectothermic animals, particularly oviparous species [5, 6]. These animals typically develop with little or no parental care and are routinely subjected to wide variations in abiotic factors such as temperature, water availability and atmospheric gases [7]. In particular, oviparous reptile nests can become severely hypoxic due to a progressive decline in nest oxygen tension from embryonic metabolism and microbial activity [8, 9]. The extent of hypoxia is nest-specific, but field estimates suggest reptilian eggs located farthest from the surface can be subjected to oxygen tensions as low as 11%, while those at the top of the nest remain at atmospheric oxygen (21%) [10]. Similar to other vertebrates, developmental hypoxia significantly alters turtle morphology and physiology, particularly at the level of the cardiovascular system [11–15]. Embryonic turtles exposed to hypoxia have different intrinsic heart rates and variable expression of receptors involved in cardiac regulation [11, 13, 16–19]. Furthermore, the effects of developmental hypoxia extend into juvenile and adult life, affecting cardiac performance and physiological traits [14, 15]. Of particular note, our recent study suggests juvenile turtles from hypoxic incubations possess cardiomyocyte specialisations that improve anoxia tolerance [20]. The programming of cardiac anoxia tolerance has major ecological implications for turtles, because many freshwater species, including *Chrysemys picta*, *Trachemys scripta*, and *Chelydra serpentina*, regularly engage in breath-hold dives that last several hours at warm temperatures, and they overwinter in anoxia for up to 5 months in ice-covered lakes [21, 22]. Even when metabolic rate and body temperature are taken into account, these freshwater turtles can survive anoxia 1000 times longer than a similarly sized mammal [23]. The maintenance of cardiac function is crucial for anoxia survival to ensure the delivery of nutrients and the removal of waste [24]. Therefore, early exposure to hypoxia may prime turtle heart physiology for a future life in anoxic environments.

The molecular mechanisms underlying cardiac programming in turtles are completely unknown but may involve epigenetic regulation of gene expression. Post-translational histone modifications and DNA methylation are the primary epigenetic marks shown to play a role in development and differentiation [25–27]. These marks regulate gene expression patterns, cell-fate decisions, and cellular physiology by altering DNA accessibility and chromatin structure. For example, trimethylation of histone H3 on lysine 4 (H3K4me3) at promoters is associated with gene activation, while trimethylation of lysine 27 on histone H3 (H3K27me3) is a repressive mark [28]. At least 70 different histone marks have been identified, each having unique effects on gene expression. The complexity of the histone code contrasts with the relative simplicity of DNA methylation, which is associated with transcriptional repression. DNA methylation is thought to inhibit transcription by interfering with transcription factor (TF) binding, though TF binding might reciprocally inhibit DNA methylation [29, 30]. Moreover, histone modifications and DNA methylation are interdependent, so de novo DNA methylation patterns laid down during embryogenesis help set the stage for maintenance of DNA methylation patterns and histone modifications through repeated cell divisions and into postnatal life [31, 32].

DNA methylation is a particularly stable, long-term mark that might be subject to environmental modification during development [33]. The most common mark is methylation of cytosines adjacent to guanines (i.e., CpG dinucleotides). Individual CpGs are typically methylated, while CpGs in clusters, called CpG islands (CGIs), are usually, though not always, found in an unmethylated state. The impact of CpG and CGI methylation on gene expression also depends upon their location within the genome. Recent work, for instance, has shown that enhancers and silencers display different patterns of CpG methylation and that orphan CGIs can act as potent enhancers [34–36]. This is on top of the classical observation that 60–70% of promoters contain CGIs [37].

Developmental hypoxia is known to alter DNA methylation and gene expression patterns in mammals, and the molecular signature is associated with cardiac abnormalities in adulthood [38, 39]. Therefore, programming of cardiac anoxia tolerance in snapping turtles may be achieved by similar mechanisms. Very little is currently known about DNA methylation landscapes in reptiles, because prior studies have almost exclusively measured global DNA methylation levels. We found one study that examined spatial patterns using MeDIP-Seq in the painted turtle, *Chrysemys picta* [40]. Key observations were that CpG distribution is bimodal in turtle promoters, as in other vertebrates [41], and that there is differential CpG methylation between hatchling ovaries and testes, including methylation differences in putative sex-determining genes. While MeDIP-Seq provides an overview of the methylation landscape at an affordable cost, it is an enrichment-based technique with shortcomings in terms of quantitatively measuring methylation levels and presenting a biased representation of the genome [42]. More importantly, we could not find a single study describing the most fundamental relationships between DNA methylation and gene expression patterns in reptiles.

In this study, we hypothesised that developmental hypoxia alters DNA methylation and gene expression patterns in turtles and that these patterns are associated with greater cardiac anoxia tolerance later in life. Snapping turtles take 9 to 18 years to reach sexual maturity, which makes it impractical to study developmental programming in adults. Instead, we tested for effects that persist in juvenile turtles months after their embryonic exposure to hypoxic conditions. To directly test these hypotheses, we first assessed cardiac performance during 2 h of acute anoxia in juvenile turtles previously exposed to normoxia (21% oxygen: N21) or hypoxia (10% oxygen: H10) during embryonic development. Next, we measured DNA methylation patterns in heart ventricles from the same cohorts using whole genome bisulfite sequencing (WGBS), the “gold standard” for DNA methylation analyses, as well as gene expression patterns using RNA-Seq. These experiments represent the first high-resolution investigation of DNA methylation patterns in any reptilian species. As hypothesized, developmental hypoxia increased juvenile turtle cardiac anoxia tolerance and programmed CpG and CGI methylation and gene expression patterns. DNA methylation and gene expression were broadly correlated at a genome-wide scale (e.g., genes with higher methylation at their promoters displayed lower expression, while those with lower promoter methylation displayed higher expression). In addition, genes that were differentially methylated between turtles from normoxic and hypoxic incubations were significantly more likely to be differentially expressed. The results suggest developmental hypoxia can programme turtle cardiovascular phenotype, spanning from molecular to physiological levels, which has important ecological implications for species that exploit anoxic environments.

## Results

### Developmental hypoxia improves cardiac anoxia tolerance

**Fig. 1:**
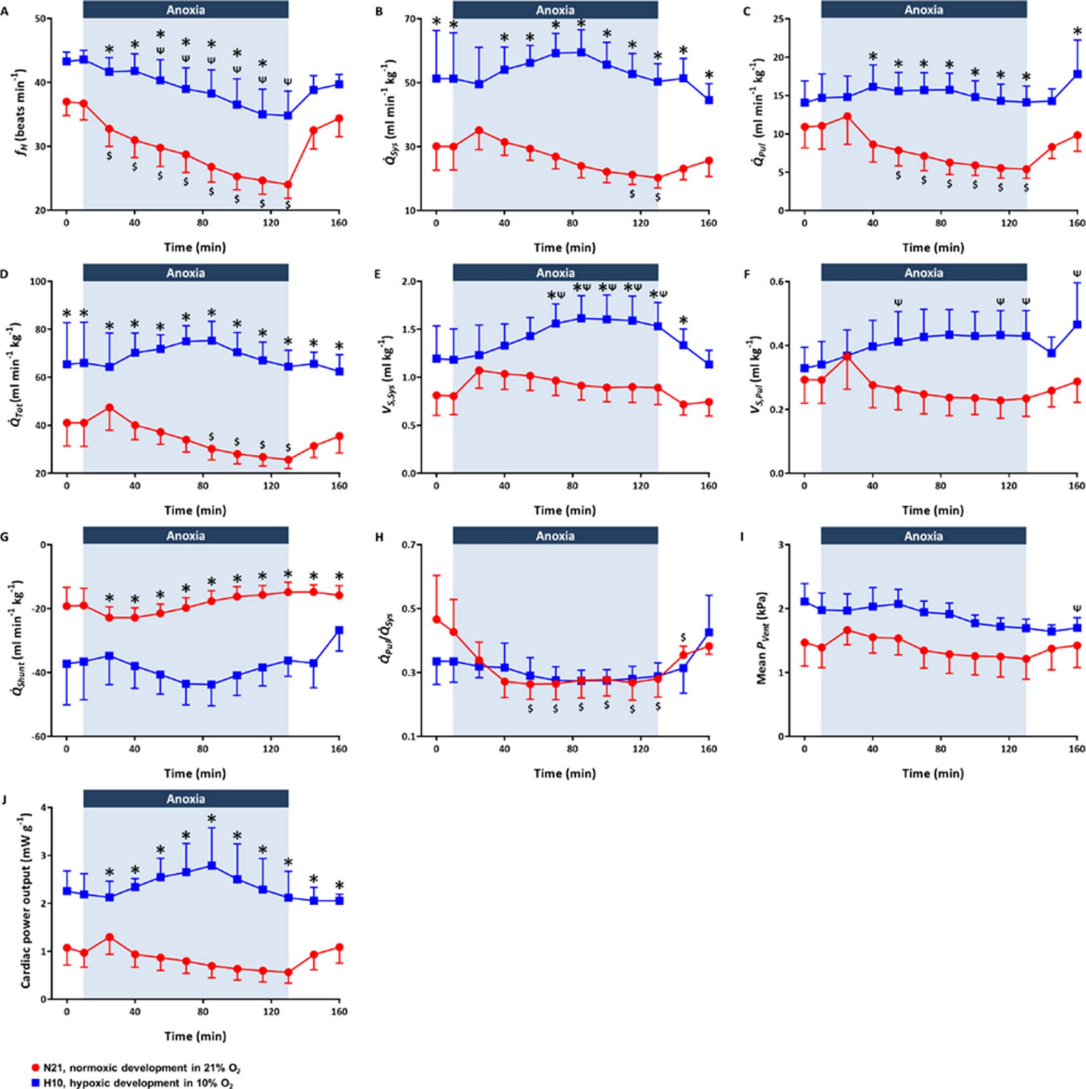
Effects of acute anoxia and reoxygenation on haemodynamic variables from N21 and H10 turtles. Turtles from the N21 (red circles, *n* = 6) and H10 (blue squares, *n* = 5) cohorts were subjected to 120 min of anoxia followed by 30 min reoxygenation. **A** Heart rate ($$f_{{\text{H}}}$$), **B** systemic blood flow ($$\dot{Q}_{{{\text{Sys}}}}$$), **C** pulmonary blood flow ($$\dot{Q}_{{{\text{Pul}}}}$$), **D** total blood flow ($$\dot{Q}_{{{\text{Tot}}}}$$), **E** systemic stroke volume ($$V_{{{\text{S}},{\text{Sys}}}}$$), **F** pulmonary stroke volume ($$V_{{{\text{S}},{\text{Pul}}}}$$), **G** shunt distribution ($$\dot{Q}_{{{\text{Shunt}}}}$$), **H** shunt ratio ($${{\dot{Q}_{{{\text{Pul}}}} } \mathord{\left/ {\vphantom {{\dot{Q}_{{{\text{Pul}}}} } {\dot{Q}_{{{\text{Sys}}}} }}} \right. \kern-\nulldelimiterspace} {\dot{Q}_{{{\text{Sys}}}} }}$$), **I** mean ventricular pressure ($$P_{{{\text{Vent}}}}$$), and **J** cardiac power output. Values are mean ± SEM, asterisks (*) indicate statistically significance difference between N21 and H10 groups, dollar ($) and psi (Ψ) symbols denote a significant difference between that data point and pre-anoxic levels (time zero) in the N21 and H10 groups, respectively (*p* ≤ 0.05)

Body and heart masses of juvenile turtles used for in situ studies of cardiovascular physiology are provided in Table 1. Acute exposure to anoxia caused a progressive bradycardia (i.e., decreased heart rate) in both experimental groups (Fig. 1A), but the magnitude of this response was significantly greater in N21 (34 ± 6%) vs. H10 (20 ± 10%) turtles. A decrease in heart rate in low oxygen environments is a key feature of the “diving reflex”, which aids in the conservation of oxygen stores in air breathing vertebrates. In the N21 group, bradycardia was associated with a progressive reduction in systemic blood flow ($\dot{Q}_{{{\text{Sys}}}}$) and pulmonary blood flow ($\dot{Q}_{{{\text{Pul}}}}$) (Fig. 1B, C), while systemic stroke volume ($V_{{{\text{S}},{\text{Sys}}}}$) and pulmonary stroke volume ($V_{{{\text{S}},{\text{Pul}}}}$) remained relatively constant (Fig. 1E, F). The reduction in pulmonary blood flow ($\dot{Q}_{{{\text{Pul}}}}$) in N21 turtles during anoxia was proportionately greater than the reduction in systemic blood flow ($\dot{Q}_{{{\text{Sys}}}}$), leading to an increase in the right-to-left (R–L) shunt of blood from the pulmonary to the systemic circulation (Fig. 1H). Turtles are able to physiologically control the outflow of blood through the pulmonary artery vs. systemic arteries (i.e., left and right aortas), because they have a three chambered heart with a single ventricle that is only partially divided by vertical and horizontal septa. An increase in R–L shunting recirculates systemic venous blood and bypasses the pulmonary circuit, while greater left-to-right (L–R) shunting recirculates blood through the pulmonary circuit. Changes in shunting may allow more efficient regulation of blood gases during periods of activity vs. rest [43]. Despite a significant reduction in total blood flow ($\dot{Q}_{{{\text{Tot}}}}$) in N21 turtles (Fig. 1D), there was only a small non-significant reduction in cardiac power output (Fig. 1J), while mean ventricular pressure remained relatively constant (Fig. 1I).

**Table 1:** Body and heart masses of juvenile snapping turtles exposed to normoxia (N21) or hypoxia (H10) during embryonic development

Cohort	Body mass (g)	Heart mass (mg)	Heart-to-body-mass ratio
N21	308.8 ± 24.5	648.7 ± 67.1	0.21 ± 0.01
H10	314.9 ± 52.2	713.4 ± 115.9	0.23 ± 0.01*

Significant differences were revealed by generalized linear models, followed by Sidak post-hoc tests, for multiple comparisons, and are denoted by asterisks (*), when *P* ≤ 0.05

**Fig. 2:**
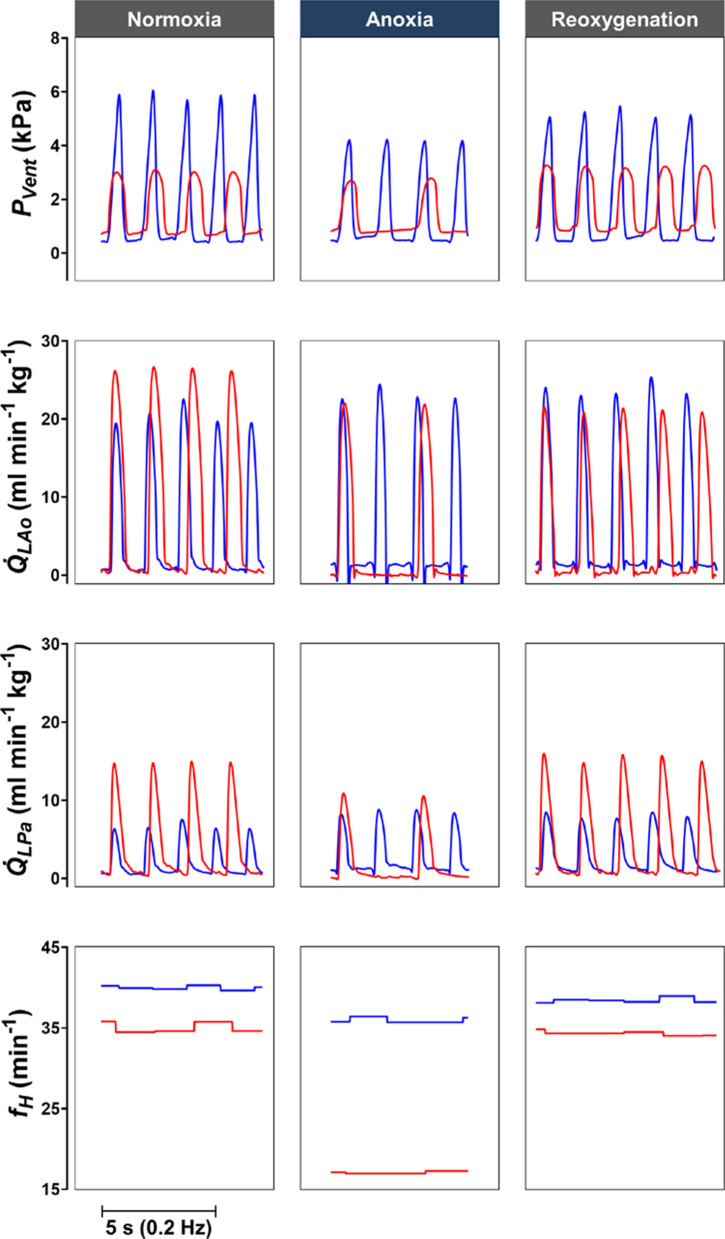
Original traces of the effects of anoxia and reoxygenation on cardiac haemodynamic variables. Ventricular pressure ($P_{{{\text{Vent}}}}$), left aortic arch blood flow ($\dot{Q}_{{{\text{LAo}}}}$), left pulmonary artery blood flow ($\dot{Q}_{{{\text{LPa}}}}$) and heart rate ($f_{{\text{H}}}$) were measured in N21 (red lines) and H10 (blue lines) turtles during 10-min normoxia, 120-min anoxia, and 20-min reoxygenation

Apart from the “diving reflex” (i.e., bradycardia), other cardiovascular responses in H10 turtles were quite distinct from N21 turtles. Surprisingly, the anoxic bradycardia in H10 turtles was not associated with any changes in systemic ($\dot{Q}_{{{\text{Sys}}}}$) or pulmonary ($\dot{Q}_{{{\text{Pul}}}}$) blood flow or the R–L shunt, which all changed in N21 turtles. This meant that systemic ($V_{{{\text{S}},{\text{Sys}}}}$) and pulmonary ($V_{{{\text{S}},{\text{Pul}}}}$) stroke volumes were significantly elevated in H10 turtles during acute anoxia (Fig. 2). As a result of the elevated stroke volume, mean ventricular pressure and cardiac power output was maintained during 2 h of anoxia in H10 turtles (Fig. 1I, J). Therefore, the H10 group maintained higher blood flows, systemic stroke volume, and heart rate ($\dot{Q}_{{{\text{Sys}}}}$, $\dot{Q}_{{{\text{Pul}}}}$, $V_{{{\text{S}},{\text{Sys}}}}$, $f_{{\text{H}}}$)and cardiac power output than the N21 cohort throughout the anoxic period (Figs. 1 and 2). In the N21 group, all haemodynamic variables reverted to normoxic levels after 30 min of reoxygenation (Fig. 1). For the H10 group, mean ventricular pressure was slightly depressed at the end of reoxygenation (Fig. 1I), and $V_{{{\text{S}},{\text{Pul}}}}$ remained elevated (Fig. 1F), while all other haemodynamic variables returned to normoxic levels (Fig. 1).

In addition to influencing responses to anoxia and reoxygenation, developmental hypoxia altered resting cardiovascular variables in snapping turtles, similar to previous reports [14]. While all haemodynamic variables fell within previously published in situ and in vivo values from *Chelydra*, *Chrysemys*, and *Trachemys* [14, 44], anaesthetised H10 turtles had significantly greater resting systemic blood flow (*Q*_Sys_)than N21 turtles, leading to a larger R–L shunt, elevated total blood flow (*Q*_Tot_) and elevated cardiac power output (Fig. 1, pre-anoxic levels). All the other haemodynamic variables were similar between experimental groups.

### Embryonic hypoxia programs transcriptome-wide patterns of gene expression

Transcriptome-wide patterns of gene expression were investigated in 7- and 9-month-old snapping turtles previously exposed to hypoxia (H10, *n* = 8) or normoxia (N21, *n* = 8) during embryonic development. Within the hypoxic cohort, turtles had two distinct cardiac phenotypes; normal-sized (*n* = 4) and enlarged (*n* = 4) hearts, relative to their body size. Gene expression within both cohorts was found to be significantly affected by age, relative heart size, and embryonic oxygen concentration. Firstly, oxygen concentration during embryogenesis altered expression of 151 genes in juvenile turtles: 75 genes were up-regulated and 76 genes were down-regulated in ventricles from the H10 group, relative to the N21 group (Table 2). Ninety-seven genes displayed significant oxygen concentration by age interactions (Table 3) and 13 of these genes were also influenced by the main effect of oxygen concentration. Finally, 256 genes were differentially expressed between ventricles from normal-sized vs. enlarged hearts (47 of these genes were among the genes listed above). A total of 131 genes were up-regulated in ventricles of enlarged hearts, while 125 genes were down-regulated (Table 4). Altogether, there were 443 differentially expressed genes.

**Table 2:** Genes that were differentially expressed between ventricles from juvenile snapping turtles exposed to normoxia (N21) or hypoxia (H10) during embryonic development

Locus #	Gene name	Gene symbol	Log_2_ (H10/N21)
CS000018264	Zinc finger family member 783		− 2.834627001
CS000001503			− 2.701680389
CS000000288	Myosin heavy chain 7		− 2.041584511
CS000024278	RALY RNA-binding protein-like	RALYL	− 1.527633917
CS000008734	Mast cell proteinase-3		− 1.496662823
CS000015120			− 1.356777695
CS000017586	Zinc finger protein 3		− 1.273941158
CS000023536	C-type lectin-domain family 2, member e		− 1.208247243
CS000009112	Calcium-binding protein 5	CABP5	− 1.181213024
CS000003610	Serine/threonine kinase 32A	STK32A	− 1.121027915
CS000017023	Adhesion G protein-coupled receptor B2		− 1.110900385
CS000011584	Tripartite motif containing 58		− 1.045883825
CS000002712	Pseudouridylate synthase 1	PUS1	− 1.035714387
CS000021405			− 1.006176746
CS000020555	Granzyme H	GZMH	− 0.853897751
CS000010063	General transcription factor IIA subunit 1-like	GTF2A1L	− 0.792335632
CS000018042	Tribbles pseudokinase 1	TRIB1	− 0.765772922
CS000008605	Contactin associated protein 1	CNTNAP1	− 0.743987654
CS000022576	Immunoglobulin heavy constant gamma 2 (G2m marker)		− 0.740245463
CS000002010	Nuclear receptor subfamily 1 group D member 2-like		− 0.731931571
CS000016349	Zinc finger protein 3		− 0.722476444
CS000011191	Deoxyribonuclease 1-like 3	DNASE1L3	− 0.710756946
CS000002882	Short chain dehydrogenase (predicted)		− 0.697002063
CS000004234	Lysozyme C-like		− 0.694683419
CS000019361	Sperm flagellar 2		− 0.637371303
CS000009788	Bone morphogenetic protein 10	BMP10	− 0.625646157
CS000022673	Centrosomal protein 295		− 0.621246238
CS000010768	MAM domain containing 4	MAMDC4	− 0.616068017
CS000002550	Family with sequence similarity 217 member B	FAM217B	− 0.606523849
CS000021864	PZP, alpha-2-macroglobulin-like		− 0.600912295
CS000022474	Dedicator of cytokinesis 2		− 0.593278276
CS000007572			− 0.564178825
CS000002388	Suppression of tumorigenicity 14	ST14	− 0.563702692
CS000010941	Coiled-coil domain containing 40	CCDC40	− 0.526253468
CS000018526	Modulator of smoothened protein	MOSMO	− 0.507345817
CS000008639	Kell blood group, metallo-endopeptidase	KEL	− 0.501729677
CS000008272			− 0.500144159
CS000013388	Coiled-coil domain containing 69		− 0.488161437
CS000023033	Calcitonin receptor	CALCR	− 0.478006366
CS000013081	PR/SET domain 8	PRDM8	− 0.470914245
CS000010506	NAD(P)H quinone dehydrogenase 2	NQO2	− 0.470257899
CS000010015	Proteolipid protein 1	PLP1	− 0.451285412
CS000009837	6-Phosphofructo-2-kinase/fructose-2,6-biphosphatase 1	PFKFB1	− 0.445995285
CS000013172	Sperm flagellar 2	SPEF2	− 0.405206127
CS000007175	Adhesion G protein-coupled receptor L3	ADGRL3	− 0.390432656
CS000018864	Zinc finger protein 3		− 0.387733152
CS000003471	DNA damage inducible transcript 4-like	DDIT4L	− 0.381244306
CS000013715	THAP domain containing 9	THAP9	− 0.367754654
CS000016980	Leucine rich repeat containing 8 family member D	LRRC8D	− 0.360578313
CS000024331	Ceramide synthase 4	CERS4	− 0.329175611
CS000004429	Diphthamide biosynthesis 7	DPH7	− 0.31321296
CS000024488	Endogenous retrovirus group MER34 member 1		− 0.283738212
CS000002360	Activating transcription factor 1	ATF1	− 0.279120084
CS000015148	Tumor necrosis factor superfamily member 10	TNFSF10	− 0.25585253
CS000001723	ATM serine/threonine kinase	ATM	− 0.249457117
CS000014235	Anthrax toxin receptor 2	ANTXR2	− 0.244822743
CS000023516	Hypoxia inducible factor 1 alpha subunit	HIF1A	− 0.237980337
CS000010455	SURF1, cytochrome c oxidase assembly factor	SURF1	− 0.222451553
CS000021833	Rho GTPase activating protein 45	ARHGAP45	− 0.221222304
CS000013956	Tribbles pseudokinase 2	TRIB2	− 0.217959675
CS000002385	Rho GTPase-activating protein 32	ARHGAP32	− 0.215929188
CS000004163	Solute carrier family 4 member 2	SLC4A2	− 0.212349304
CS000010124	Solute carrier family 35 member A1	slc35a1	− 0.20854517
CS000007017	Cingulin-like 1	CGNL1	− 0.198504808
CS000009705	F-box and leucine rich repeat protein 20	FBXL20	− 0.168085068
CS000025011	Retinoic acid receptor, alpha		0.154424605
CS000009251	Taxilin beta	TXLNB	0.158953455
CS000005867	Epidermal growth factor receptor pathway substrate 8	EPS8	0.163637442
CS000023373	Mannosidase beta	MANBA	0.166087668
CS000021122	Sprouty related EVH1 domain containing 2	SPRED2	0.170050721
CS000008545	Erb-b2 receptor tyrosine kinase 2	ERBB2	0.187176414
CS000000599	Tubulin folding cofactor B	TBCB	0.195969098
CS000013318	G protein subunit gamma 10	GNG10	0.208058965
CS000003073	Cadherin 11, type 2, OB-cadherin (osteoblast)		0.208906093
CS000008574	2′,3′-Cyclic nucleotide 3′ phosphodiesterase	CNP	0.212460195
CS000007985	STARD3 N-terminal-like	STARD3NL	0.219747393
CS000009739	Tropomyosin 3	TPM3	0.236963141
CS000024894	Serine/threonine kinase 38-like	STK38L	0.238422059
CS000015359	Spectrin beta, erythrocytic		0.241602606
CS000013674	Eva-1 homolog C	EVA1C	0.245944042
CS000010784	Cholesteryl ester transfer protein	CETP	0.246380083
CS000010497	Serpin peptidase inhibitor, clade B (ovalbumin), member 6		0.248181093
CS000009948			0.252507277
CS000008411	Integrin subunit alpha 11	ITGA11	0.254329861
CS000003646	Ubiquitin conjugating enzyme E2 B	UBE2B	0.254817922
CS000014028	C-type lectin-domain family 2 member D		0.264505749
CS000011229	Ribonuclease H2 subunit C	rnaseh2c	0.27146634
CS000023524	Protein tyrosine phosphatase, non-receptor type 23		0.277562695
CS000009501	Heparan sulfate 6-*O*-sulfotransferase 2	HS6ST2	0.28065352
CS000005400	Inositol-trisphosphate 3-kinase A	ITPKA	0.29921558
CS000008774	Mitogen-activated protein kinase kinase kinase 5	MAP3K5	0.309585444
CS000019402	Heat shock protein family B (small) member 3	HSPB3	0.328843966
CS000014776	Bardet-Biedl syndrome 1 protein		0.33633198
CS000004507	Cathepsin L	ctsl	0.344555132
CS000013316	KIAA0368	KIAA0368	0.353983994
CS000020738	Protein phosphatase, Mg^2+^/Mn^2+^-dependent 1H	PPM1H	0.35982697
CS000010357	Potassium voltage-gated channel subfamily H member 6	KCNH6	0.370923213
CS000000947	Pleckstrin homology-like domain family A member 3	PHLDA3	0.371384191
CS000000189	Protein kinase AMP-activated non-catalytic subunit beta 2	PRKAB2	0.376457859
CS000021606	Ependymin related 1	EPDR1	0.384457752
CS000013259	Centromere protein H	CENPH	0.384685483
CS000004917	Bone morphogenetic protein receptor type 2	BMPR2	0.400771813
CS000008544	Growth factor receptor bound protein 7	GRB7	0.443902422
CS000001051	Monooxygenase DBH-like 1	MOXD1	0.459634006
CS000003463	Alcohol dehydrogenase 4 (class II), pi polypeptide	ADH4	0.494122016
CS000017173	Solute carrier family 2 member 11	SLC2A11	0.501060861
CS000011090	Secreted phosphoprotein 1	SPP1	0.517276571
CS000021119	Endogenous retrovirus group MER34 member 1		0.524982887
CS000014447	PDZ-binding kinase	PBK	0.542088418
CS000011287	Dual specificity protein phosphatase 10-like		0.543863344
CS000009633	Activated leukocyte cell adhesion molecule	ALCAM	0.548683718
CS000010233	EFR3 homolog B		0.554021781
CS000001795	Transmembrane protein 71	TMEM71	0.60589964
CS000001243	Myelin basic protein	MBP	0.606334331
CS000012858	Suppressor of cytokine signaling 2	SOCS2	0.635342108
CS000001161	Transmembrane protein 200C	tmem200c	0.646446549
CS000020994	Transmembrane protein 151B-like		0.64955796
CS000004537	Charged multivesicular body protein 4C	CHMP4C	0.649900032
CS000008619	ETS variant 4	ETV4	0.668839089
CS000014855	Killer cell lectin-like receptor subfamily G, member 2		0.669227747
CS000018990	Collagen type XXII alpha 1 chain	COL22A1	0.675489915
CS000019220	Keratin 8	KRT8	0.683602204
CS000019009	Thrombospondin type 1 domain containing 7A	THSD7A	0.718432909
CS000005131	Glutaredoxin	GLRX	0.740909237
CS000019856	TNF receptor associated factor 2		0.756354667
CS000004598			0.765230508
CS000006964	Adaptor related protein complex 1 sigma 3 subunit	AP1S3	0.765814686
CS000021260	Potassium voltage-gated channel subfamily A member 4	KCNA4	0.854709853
CS000003440	Apolipoprotein C1	apoc1	0.878140809
CS000019191	Interaction protein for cytohesin exchange factors 1	IPCEF1	0.879401672
CS000009567	Keratin 18	KRT18	0.925644492
CS000022727	Fer-1-like family member 4	FER1L4	0.964212443
CS000020607	DNA polymerase nu	POLN	1.00608115
CS000018642	Hyaluronan-binding protein 2	HABP2	1.034969644
CS000014808	Synaptonemal complex protein 1	SYCP1	1.124476724
CS000004949	Myosin light chain 1	MYL1	1.168968857
CS000011884	Ubiquinol-cytochrome c reductase complex assembly factor 2		1.298675033
CS000004501			1.320990056
CS000004709	Heparan sulfate–glucosamine 3-sulfotransferase 2	HS3ST2	1.353228055
CS000009646	Adhesion G protein-coupled receptor G7	ADGRG7	1.423863767
CS000001877	Glycoprotein nmb	GPNMB	1.608451449
CS000012011	Coagulation factor III, tissue factor	F3	1.645631227
CS000004554	Complement C1r	C1R	1.873883015
CS000000330	Matrix metallopeptidase 25	MMP25	1.977548442
CS000012602	Neuritin 1	NRN1	2.508897571
CS000019631	Perforin 1		2.667492969
CS000021046	Complement C1r subcomponent		2.710546811
CS000013720	Vesicle-associated membrane protein 8-like		3.126479237
CS000010724	LIM homeobox 5	LHX5	3.176148053
CS000018878			4.264821788
CS000012122	Astacin-like metalloendopeptidase		4.556056058

The difference in expression in the last column is calculated as the log_2_ of the ratio of gene expression in turtles exposed to hypoxia divided by gene expression in turtles exposed to normoxia during embryonic development. Negative values indicate the gene was downregulated in the hypoxic group, while positive values indicate the gene was upregulated in the hypoxic group. The transcriptome was analyzed via RNA-Seq. Differences in gene expression were considered significant when results from DESeq2 and ANOVA were concordant

**Table 3:** Genes that displayed significant oxygen concentration by age interactions in ventricles from juvenile snapping turtles exposed to normoxia (N21) or hypoxia (H10) during embryonic development and sampled at 7 months or 9 months of age

locus_number	gene_name	gene_symbol
CS000013624	von Willebrand factor A domain containing 5B1	VWA5B1
CS000017075	Adhesion G protein-coupled receptor D2	ADGRD2
CS000002650	Doublecortin domain containing 1	
CS000020386		
CS000005701	Extracellular leucine rich repeat and fibronectin type III domain containing 1	ELFN1
CS000007756	Raf-1 proto-oncogene, serine/threonine kinase	RAF1
CS000012404		
CS000011576	Immunity related GTPase cinema	
CS000017702		
CS000017791	Gag-pol precursor polyprotein	
CS000005252	Myomesin 3	MYOM3
CS000013643	Heat shock protein 30C L homeolog	
CS000007769		
CS000000238	Tryptophan hydroxylase 1	TPH1
CS000022856		
CS000011558	Transmembrane channel-like 5	TMC5
CS000020596	Sortilin related VPS10 domain containing receptor 2	SORCS2
CS000004923	CD28 molecule	CD28
CS000008982	Regulator of G-protein signaling 5	RGS5
CS000001877	Glycoprotein nmb	GPNMB
CS000011960	Crystallin alpha B	CRYAB
CS000000625	HEN1 methyltransferase homolog 1	HENMT1
CS000011913	Heat shock protein 30C L homeolog	
CS000021013	Fibrous sheath interacting protein 1	FSIP1
CS000005377	Galectin 1	LGALS1
CS000017153	Guanylate-binding protein 2-like	
CS000000187	Phosphoglycerate dehydrogenase	PHGDH
CS000011024	Target of myb1-like 1 membrane trafficking protein	TOM1L1
CS000016415	Endogenous retrovirus group V member 2	
CS000011191	Deoxyribonuclease 1 like 3	DNASE1L3
CS000003395	Ankyrin repeat and SOCS box containing 18	ASB18
CS000004493	Spindle assembly abnormal protein 6 homolog	
CS000024997	Toll like receptor 6	TLR6
CS000023735		
CS000001051	Monooxygenase DBH like 1	MOXD1
CS000000229	Potassium voltage-gated channel subfamily J member 11	KCNJ11
CS000001730	Ferredoxin 1	FDX1
CS000005131	Glutaredoxin	GLRX
CS000006114	Chordin like 1	CHRDL1
CS000012086	Actin, aortic smooth muscle-like	
CS000003405	Fibronectin 1	FN1
CS000003059	Carboxylesterase 2	CES2
CS000003128	Cytochrome b-245 alpha chain	CYBA
CS000000901	Transmembrane protein 159 L homeolog	tmem159.L
CS000023152	Mindbomb E3 ubiquitin protein ligase 1	MIB1
CS000017081	Prostaglandin-endoperoxide synthase 1	PTGS1
CS000017005	Muskelin 1	MKLN1
CS000004866	Myosin IB	MYO1B
CS000013316	KIAA0368	KIAA0368
CS000001871	Rap guanine nucleotide exchange factor 5	RAPGEF5
CS000015369	Acyl-CoA-binding domain containing 6	ACBD6
CS000019402	Heat shock protein family B (small) member 3	HSPB3
CS000004507	Cathepsin L	ctsl
CS000009267	RAB32, member RAS oncogene family	RAB32
CS000003646	Ubiquitin conjugating enzyme E2 B	UBE2B
CS000023259	Crystallin zeta	CRYZ
CS000013749	Pitrilysin metallopeptidase 1	PITRM1
CS000024623	Myosin VC	MYO5C
CS000009274	Glycoprotein integral membrane 1	GINM1
CS000008916	Transmembrane protein 214	TMEM214
CS000002191	Endothelin-converting enzyme 2	ECE2
CS000002902	Gamma-aminobutyric acid type B receptor subunit 1	GABBR1
CS000011567	Glycogen synthase 1	GYS1
CS000024960	DExH-box helicase 9	DHX9
CS000023516	Hypoxia inducible factor 1 alpha subunit	HIF1A
CS000009882	Acyl-CoA oxidase 1	ACOX1
CS000017504	Unc-5 netrin receptor A	UNC5A
CS000005022	DnaJ heat shock protein family (Hsp40) member C11	DNAJC11
CS000003466	tRNA methyltransferase 10A	TRMT10A
CS000001723	ATM serine/threonine kinase	ATM
CS000007175	Adhesion G protein-coupled receptor L3	ADGRL3
CS000018314	General transcription factor IIIC subunit 2	
CS000003458	SWI/SNF-related, matrix-associated actin-dependent regulator of chromatin, subfamily a, containing DEAD/H box 1	SMARCAD1
CS000015974	Coiled-coil domain containing 130	CCDC130
CS000009858	Arginine demethylase and lysine hydroxylase	JMJD6
CS000025151	Jumonji and AT-rich interaction domain containing 2	JARID2
CS000014359	Peroxisomal biogenesis factor 26	PEX26
CS000004897	Transmembrane protein 237	TMEM237
CS000014907	Zinc finger protein 219	ZNF219
CS000014046	G protein-coupled receptor kinase 5-like	
CS000011309	Zinc finger and SCAN domains 20	
CS000003047	WD repeat domain 88	WDR88
CS000003006	Mucolipin 3	MCOLN3
CS000008630		
CS000007667	FXYD domain containing ion transport regulator 3	
CS000003008	Mucolipin 2	MCOLN2
CS000019541	Chromosome 21 orf 58	
CS000009617	Transmembrane protease, serine 7	TMPRSS7
CS000019987	Plexin A3	
CS000020555	Granzyme H	GZMH
CS000009788	Bone morphogenetic protein 10	BMP10
CS000011540	von Willebrand factor A domain containing 3A	VWA3A
CS000021466		
CS000015152		
CS000008845	Apolipoprotein F	APOF
CS000023912		
CS000020445	UPF0061 protein xcc-b100,1894-like	

The transcriptome was analyzed via RNA-Seq. Differences in gene expression were considered significant when results from DESeq2 and ANOVA were concordant

**Table 4:** Genes that were differentially expressed between ventricles from juvenile snapping turtles that had normal-sized or enlarged hearts relative to their body size

Locus #	Gene name	Gene symbol	Log_2_ (enlarged/normal)
CS000003025	DnaJ heat shock protein family (Hsp40) member A2	DNAJA2	− 4.00011659
CS000005942	Mucin 2, oligomeric mucus/gel-forming	MUC2	− 4
CS000020789			− 4
CS000020715			− 3.970542981
CS000000246			− 3.412275459
CS000000714	Interleukin 22	IL22	− 2.460702344
CS000008734	Mast cell proteinase-3		− 2.313632939
CS000006768	Capping protein, Arp2/3 and myosin-I linker protein 3-like		− 2.266058389
CS000018994	Capping protein regulator and myosin 1 linker 3	CARMIL3	− 2.256232917
CS000008168	Glucagon	GCG	− 1.725877497
CS000002882	Short chain dehydrogenase (predicted)		− 1.68846675
CS000020555	Granzyme H	GZMH	− 1.641521247
CS000011467	T brachyury transcription factor	TBXT	− 1.62277543
CS000009580	Advillin	AVIL	− 1.59555768
CS000017383	G protein-coupled receptor 62	GPR62	− 1.591541521
CS000018663	Deleted in malignant brain tumors 1 protein-like		− 1.525993727
CS000008605	Contactin associated protein 1	CNTNAP1	− 1.459617958
CS000010544	C-type lectin-domain family 2 member D	CLEC2D	− 1.349128576
CS000014353	Aldo–keto reductase family 1, member C3 (3-alpha hydroxysteroid dehydrogenase, type II)		− 1.341939485
CS000008622	Sclerostin	sost	− 1.338528092
CS000013408	Solute carrier family 8 member A2	SLC8A2	− 1.333783678
CS000012528	Persephin	PSPN	− 1.322706586
CS000009180	CD69 molecule	CD69	− 1.320171193
CS000019803			− 1.292382379
CS000000790	NFKB inhibitor like 1	NFKBIL1	− 1.268443921
CS000020022	Carcinoembryonic antigen related cell adhesion molecule 1	CEACAM1	− 1.249402634
CS000009788	Bone morphogenetic protein 10	BMP10	− 1.185425524
CS000012770	Nudix hydrolase 15	NUDT15	− 1.174821415
CS000002010	Nuclear receptor subfamily 1 group D member 2-like	NR1D2	− 1.135755617
CS000012951	Zinc finger CW-type and PWWP domain containing 1	ZCWPW1	− 1.131146467
CS000015104	Rho GTPase activating protein 27	ARHGAP27	− 1.12138132
CS000009000	Cytochrome P450 family 8 subfamily B member 1	CYP8B1	− 1.119197734
CS000023981	Zinc finger protein 501-like		− 1.109988843
CS000019117			− 1.093528795
CS000004600			− 1.076659475
CS000020803			− 1.076581749
CS000024876	MHC class II DLA DRB1 beta chain		− 0.99585363
CS000003266	Synaptotagmin-15	SYT15	− 0.992965859
CS000013911			− 0.970276375
CS000022474	Dedicator of cytokinesis 2	DOCK2	− 0.970046644
CS000011784	C-type lectin-domain family 2, member e		− 0.947959012
CS000009177	Zinc finger protein 2	ZNF2	− 0.9470744
CS000017654	Junctophilin 4	JPH4	− 0.9449115
CS000017263	USH1 protein network component harmonin	USH1C	− 0.941279454
CS000012524	Synovial sarcoma, X breakpoint 2 interacting protein S homeolog	ssx2ip.S	− 0.939268547
CS000000565	Ras and Rab interactor-like protein	RINL	− 0.892810687
CS000023033	Calcitonin receptor	CALCR	− 0.88702029
CS000016033	Ring finger protein 25	rnf25	− 0.882108694
CS000021269	Neural EGFL like 1	NELL1	− 0.868462024
CS000012971	Neuron derived neurotrophic factor	NDNF	− 0.866800964
CS000020495	Kinase non-catalytic C-lobe domain containing 1	KNDC1	− 0.852740249
CS000002530	Collagen type XX alpha 1 chain	COL20A1	− 0.843443212
CS000012304			− 0.799063985
CS000001767	Septin 5	SEPTIN5	− 0.795109503
CS000013081	PR/SET domain 8	PRDM8	− 0.778512974
CS000024963	Zinc finger protein 862	ZNF862	− 0.776513766
CS000002362	Acid sensing ion channel subunit 1	asic1	− 0.77548223
CS000006802	Sortilin related VPS10 domain containing receptor 1	SORCS1	− 0.774579369
CS000003991	Nuclear receptor subfamily 0 group B member 2-like	NR0B2	− 0.752752113
CS000008630			− 0.742661864
CS000011782	C-type lectin-domain family 2 member D	CLEC2D	− 0.738112094
CS000020166	Tweety family member 1	TTYH1	− 0.727823227
CS000017666	Spectrin beta, non-erythrocytic 4	SPTBN4	− 0.727759339
CS000019541	Chromosome 21 orf 58	C21orf58	− 0.693870417
CS000014966	Ficolin 3	FCN3	− 0.683027443
CS000007237	Collectin subfamily member 10	COLEC10	− 0.67828483
CS000023359	Ral guanine nucleotide dissociation stimulator like 3	RGL3	− 0.676871711
CS000001261			− 0.673565294
CS000015636	Ecto-NOX disulfide-thiol exchanger 1	enox1	− 0.667186029
CS000018597	Neurexin 2	NRXN2	− 0.658378249
CS000004183	Kelch-like 42		− 0.645354432
CS000005642	Topoisomerase (DNA) III alpha	TOP3A	− 0.637881286
CS000012953	Immunoglobulin superfamily containing leucine-rich repeat	ISLR	− 0.636348281
CS000001835	Actin-binding Rho activating protein	ABRA	− 0.622678244
CS000006362	Nicotinamide riboside kinase 2	NMRK2	− 0.57894428
CS000000038	KH and NYN domain containing	KHNYN	− 0.560880734
CS000004606	Aryl hydrocarbon receptor nuclear translocator like	ARNTL	− 0.550619038
CS000008808	Alcohol dehydrogenase, iron containing 1	ADHFE1	− 0.546993977
CS000012473	Progestin and adipoQ receptor family member 6	PAQR6	− 0.540783679
CS000020112	Ral guanine nucleotide dissociation stimulator-like 2	RGL2	− 0.528180794
CS000024555	Stimulator of chondrogenesis 1	SCRG1	− 0.525168151
CS000003254	Tubulin tyrosine ligase like 3	TTLL3	− 0.517188122
CS000010967			− 0.516398171
CS000002912	Tripartite motif containing 21	TRIM21	− 0.514703
CS000016053	Sosondowah ankyrin repeat-domain family member C	SOWAHC	− 0.50414698
CS000007586	Proline and arginine rich end leucine rich repeat protein	PRELP	− 0.495350825
CS000019628	Predicted gene 4070		− 0.478962063
CS000000139	Galactosidase beta 1 like	glb1l	− 0.476795916
CS000009979	Cysteine rich transmembrane BMP regulator 1	CRIM1	− 0.465661576
CS000025399	Collagen type IV alpha 6 chain	COL4A6	− 0.464490703
CS000014745	Cadherin EGF LAG seven-pass G-type receptor 3	celsr3	− 0.458822636
CS000002394	Neurotrimin	NTM	− 0.456792451
CS000024488	Endogenous retrovirus group MER34 member 1	ERVMER34-1	− 0.456570624
CS000002168	fibroblast growth factor receptor 3	FGFR3	− 0.45517114
CS000009429	Endogenous retrovirus group PABLB member 1 Env polyprotein	ERVPABLB-1	− 0.454624962
CS000000163	DNA polymerase kappa	POLK	− 0.449829124
CS000000791	TNF receptor-associated factor 2-like		− 0.446715719
CS000013923			− 0.438881138
CS000010076	Tripartite motif containing 27	TRIM27	− 0.437359644
CS000012780	Potassium channel regulator	KCNRG	− 0.435061589
CS000020545	Nicotinamide N-methyltransferase-like		− 0.425412793
CS000018595	Signal-induced proliferation-associated 1	SIPA1	− 0.420335966
CS000017396	RNA-binding motif protein 5	RBM5	− 0.420294548
CS000015645	Regulator of cell cycle	RGCC	− 0.410119835
CS000008765	TBC1 domain containing kinase	TBCK	− 0.409881882
CS000010919	Solute carrier family 25 member 10	SLC25A10	− 0.402643235
CS000002886	Collagen type XV alpha 1 chain	COL15A1	− 0.399950238
CS000013998	Patatin like phospholipase domain containing 7	PNPLA7	− 0.393493799
CS000009498	Glypican 3	GPC3	− 0.383165019
CS000025210	Wnt family member 11	WNT11	− 0.382187878
CS000013883	BCL tumor suppressor 7B	BCL7B	− 0.378585114
CS000023516	Hypoxia inducible factor 1 alpha subunit	HIF1A	− 0.377701984
CS000014058	Collagen, type XVIII, alpha 1	COL18A1	− 0.360702793
CS000017272	RNA-binding motif protein 10	RBM10	− 0.359302205
CS000001770	Neuroepithelial cell transforming 1	NET1	− 0.352443733
CS000004853	Collagen type III alpha 1 chain	COL3A1	− 0.328008574
CS000024953			− 0.321005944
CS000005987	Tyrosine kinase 2	TYK2	− 0.298269494
CS000012361	FUS RNA-binding protein	FUS	− 0.29508553
CS000007770	Nucleolar protein 8	NOL8	− 0.294633032
CS000024095	Syntrophin beta 1	SNTB1	− 0.294318957
CS000003363	Programmed cell death 11	PDCD11	− 0.264560467
CS000018465	Family with sequence similarity 234 member A	FAM234A	− 0.255178537
CS000007341	Catenin delta 1	CTNND1	− 0.251866178
CS000012875	LOC400927-CSNK1E readthrough	TPTEP2-CSNK1E	− 0.230967711
CS000003886	PDZ domain containing ring finger 3	PDZRN3	0.22442223
CS000020331	Cyclin-dependent kinase 17	CDK17	0.241724877
CS000008574	2′,3′-Cyclic nucleotide 3′ phosphodiesterase	CNP	0.266261699
CS000015509	Sodium voltage-gated channel alpha subunit 5	SCN5A	0.310625656
CS000009739	Tropomyosin 3	TPM3	0.312184844
CS000025418	Peptidylprolyl isomerase A	PPIA	0.314495662
CS000023518	MNAT1, CDK activating kinase assembly factor	MNAT1	0.323120508
CS000013900	*N*-Acetylglucosamine-1-phosphate transferase alpha and beta subunits	GNPTAB	0.324699426
CS000008711	Synaptosome associated protein 29	SNAP29	0.325626966
CS000004194	Mitochondrial ribosomal protein S18C	MRPS18C	0.327277429
CS000008823	Nuclear receptor coactivator 2	NCOA2	0.327760834
CS000002566	VAMP associated protein B and C	VAPB	0.327878453
CS000023702	Prefoldin subunit 1	PFDN1	0.332371862
CS000008476	Growth factor receptor bound protein 10	GRB10	0.338931617
CS000004780	Amyloid beta precursor protein-binding family A member 1	APBA1	0.340118016
CS000006903	Family with sequence similarity 183, member A	FAM183A	0.340987831
CS000001976	HAUS augmin like complex subunit 8	HAUS8	0.3445911
CS000012782	SPRY domain containing 7	SPRYD7	0.353656911
CS000011256	Sushi domain containing 3	SUSD3	0.362482561
CS000013749	Pitrilysin metallopeptidase 1	PITRM1	0.38762199
CS000007925	Family with sequence similarity 118 member B	FAM118B	0.392163014
CS000020039	Mitochondrial intermediate peptidase	MIPEP	0.394441129
CS000014649	Hematological and neurological expressed 1	HN1	0.40118652
CS000003093	Cadherin 13	CDH13	0.40428373
CS000008885	Neural precursor cell expressed, developmentally down-regulated 9	NEDD9	0.406086208
CS000009999	HAUS augmin like complex subunit 1	HAUS1	0.407190489
CS000012325	Receptor interacting serine/threonine kinase 2	RIPK2	0.407834454
CS000003646	Ubiquitin conjugating enzyme E2 B	UBE2B	0.409403598
CS000015494	Limb development membrane protein 1	LMBR1	0.413015468
CS000014842	LARGE xylosyl- and glucuronyltransferase 1	LARGE1	0.418322361
CS000009751	Myosin light chain 7	MYL7	0.419170988
CS000003679	Mitogen-activated protein kinase 9	MAPK9	0.422947717
CS000000944	Troponin T2, cardiac type	TNNT2	0.431038749
CS000002295	Family with sequence similarity 83 member H	fam83h	0.431750631
CS000007019	Aquaporin 9	AQP9	0.436004498
CS000016136	Early endosome antigen 1	EEA1	0.443580231
CS000023302	Cdc42 guanine nucleotide exchange factor 9	ARHGEF9	0.459948169
CS000021606	Ependymin related 1	EPDR1	0.477676177
CS000011866	Ras homolog family member C	RHOC	0.481673372
CS000019402	Heat shock protein family B (small) member 3	HSPB3	0.490224239
CS000013608	Transmembrane protein 51	TMEM51	0.490548583
CS000023885	Formin homology 2 domain containing 3	FHOD3	0.495382703
CS000018274	Cysteine and glycine rich protein 1	CSRP1	0.498739165
CS000003393	ArfGAP with GTPase domain, ankyrin repeat and PH domain 1	AGAP1	0.499342757
CS000002632	Cysteine and glycine rich protein 3	CSRP3	0.506402578
CS000023152	Mindbomb E3 ubiquitin protein ligase 1	MIB1	0.506464289
CS000005359	Synaptotagmin 11	SYT11	0.507330807
CS000000947	Pleckstrin homology-like domain family A member 3	PHLDA3	0.523937739
CS000000229	Potassium voltage-gated channel subfamily J member 11	KCNJ11	0.524801955
CS000020271	Myosin, light chain 12A, regulatory, non-sarcomeric	MYL12A	0.529612298
CS000011198	Adaptor protein, phosphotyrosine interacting with PH domain and leucine zipper 1	APPL1	0.535485261
CS000000757	Testis development related protein	TDRP	0.552047882
CS000005687	G protein subunit alpha 12	GNA12	0.556060953
CS000000925	Nicotinamide riboside kinase 2-like		0.559479152
CS000020993	TYRO3 protein tyrosine kinase	TYRO3	0.560184602
CS000009817	Rho related BTB domain containing 2	RHOBTB2	0.561224935
CS000011090	Secreted phosphoprotein 1	SPP1	0.562276569
CS000006432	Glutathione peroxidase 4	GPX4	0.570135795
CS000000499	Myopalladin	MYPN	0.570884545
CS000007378	Heat shock protein family B (small) member 1	HSPB1	0.57397187
CS000012962	Protease, serine 12	PRSS12	0.583446299
CS000005505	SIVA1 apoptosis inducing factor	SIVA1	0.584199204
CS000023280	Sorbin and SH3 domain containing 2	SORBS2	0.592192854
CS000008152	LY75-CD302 readthrough	LY75-CD302	0.601643015
CS000006114	Chordin like 1	CHRDL1	0.606688517
CS000005009	Ring finger protein 207	RNF207	0.606887706
CS000004917	Bone morphogenetic protein receptor type 2	BMPR2	0.608134843
CS000008531	Digestive cysteine proteinase 2-like		0.608312906
CS000000963	Cardiac-enriched FHL2-interacting protein	CUNH10orf71	0.619320903
CS000020738	Protein phosphatase, Mg^2+^/Mn^2+^-dependent 1H	PPM1H	0.625433587
CS000001051	Monooxygenase DBH like 1	MOXD1	0.627515741
CS000019311	Protein kinase C alpha	PRKCA	0.628980813
CS000010890	Family with sequence similarity 169 member A	FAM169A	0.647101757
CS000023075	CD109 molecule	CD109	0.649459241
CS000005076			0.653243213
CS000014425	B-Raf proto-oncogene, serine/threonine kinase	BRAF	0.65486808
CS000004507	Cathepsin L	ctsl	0.655047104
CS000012086	Actin, aortic smooth muscle-like	ACTA2	0.670113468
CS000013316	KIAA0368	KIAA0368	0.692998191
CS000018070	Male germ cell associated kinase	MAK	0.700509414
CS000012890	Sestrin 3	SESN3	0.706989256
CS000003989	Pleckstrin homology domain interacting protein	PHIP	0.710494062
CS000017173	Solute carrier family 2 member 11	SLC2A11	0.732703259
CS000020613	Ribosomal protein S18-like		0.73789984
CS000009633	Activated leukocyte cell adhesion molecule	ALCAM	0.742827537
CS000011644	DNA polymerase zeta catalytic subunit	REV3L	0.770984303
CS000004444	RAB33A, member RAS oncogene family	RAB33A	0.780890859
CS000014388	BICD cargo adaptor 1	BICD1	0.789743481
CS000019543			0.821651965
CS000009594	UDP glycosyltransferase 8-like	UGT8L	0.823852981
CS000008337	Protein phosphatase, Mg^2+^/Mn^2+^-dependent 1L	PPM1L	0.848557469
CS000009656	Collagen type VIII alpha 1 chain	COL8A1	0.897700653
CS000006655	G protein-coupled receptor kinase 3	GRK3	0.898389538
CS000012858	Suppressor of cytokine signaling 2	SOCS2	0.945191217
CS000010981	ATP-binding cassette subfamily A member 5	ABCA5	0.957982052
CS000001795	Transmembrane protein 71	TMEM71	0.991430672
CS000023496	AHNAK nucleoprotein 2	AHNAK2	0.996386481
CS000003395	Ankyrin repeat and SOCS box containing 18	ASB18	1.000007423
CS000019856	TNF receptor associated factor 2	TRAF2	1.000486113
CS000004132	Low density lipoprotein receptor class A domain containing 4	LDLRAD4	1.016079793
CS000018434			1.023357895
CS000021605	Secreted frizzled related protein 4	SFRP4	1.024078897
CS000004537	Charged multivesicular body protein 4C	CHMP4C	1.033646338
CS000009567	Keratin 18	KRT18	1.107725712
CS000018802	LanC like 3	LANCL3	1.116482677
CS000005377	Galectin 1	LGALS1	1.139460825
CS000014226	MOB kinase activator 1B	MOB1B	1.171832671
CS000018642	Hyaluronan-binding protein 2	HABP2	1.185512417
CS000006855	Transforming acidic coiled-coil-containing protein 2	TACC2	1.237644425
CS000005131	Glutaredoxin	GLRX	1.274526454
CS000022717	Fibrinogen C domain containing 1	FIBCD1	1.354378958
CS000024186	Leucine rich repeat containing 3B	LRRC3B	1.366458011
CS000011960	Crystallin alpha B	CRYAB	1.397658976
CS000013643	Heat shock protein 30C L homeolog		1.419468073
CS000018346	Abnormal spindle microtubule assembly	ASPM	1.420184199
CS000014980	Complement factor B	CFB	1.459428513
CS000011910	Heat shock protein 30C L homeolog		1.508403744
CS000008585	Heat shock protein, alpha-crystallin-related, b11		1.521530969
CS000005252	Myomesin 3	MYOM3	1.56777953
CS000012224	TNF receptor associated factor 3	TRAF3	1.605321051
CS000021043	Complement C1s	C1S	1.630815717
CS000013370	Ectonucleoside triphosphate diphosphohydrolase 3	entpd3	1.743604465
CS000004501			1.793715013
CS000007782	Musculoskeletal, embryonic nuclear protein 1	mustn1	1.852375511
CS000022000	Mast cell proteinase-1	CPT1B	1.895535655
CS000016407	Fc fragment of IgG-binding protein	FCGBP	1.955167136
CS000012089	CD59 Molecule (CD59 Blood Group)-like	CD59	2.005971296
CS000001877	Glycoprotein nmb	GPNMB	2.090003205
CS000025123	Mast cell proteinase-3		2.432617325
CS000000238	Tryptophan hydroxylase 1	TPH1	2.525652753
CS000006963	Secretogranin II	SCG2	3.207453439

The difference in expression in the last column is calculated as the log_2_ of the ratio of gene expression in enlarged hearts divided by gene expression in normal-sized hearts. Negative values indicate the gene was downregulated in enlarged hearts, while positive values indicate the gene was upregulated in enlarged hearts. The transcriptome was analyzed via RNA-Seq. Differences in gene expression were considered significant when results from DESeq2 and ANOVA were concordant

**Fig. 3:**
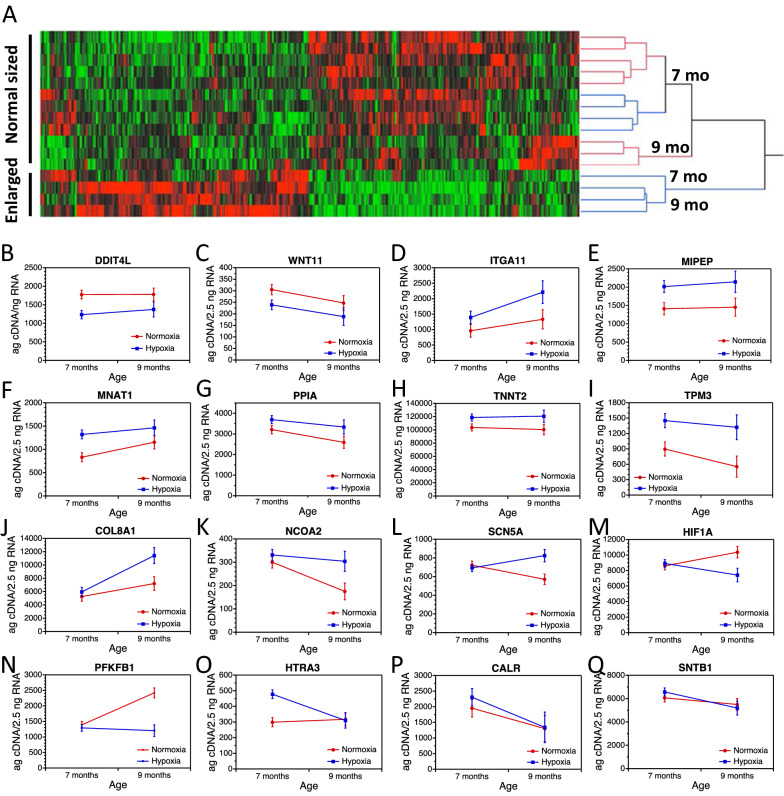
Gene expression patterns in ventricles of 7-month-old and 9-month-old snapping turtles that had been incubated in normoxic or hypoxic conditions as embryos. Samples from the N21 cohort are shown with red circles and red lines, while the H10 cohort is shown with blue circles and blue lines. **A** Heatmap of RNA-Seq expression values for the 443 genes that were significantly affected by oxygen concentration during embryogenesis (Table 2), the oxygen concentration by age interaction (Table 3), and/or differed between ventricles from juvenile snapping turtles that had normal-sized or enlarged hearts relative to their body size (Table 4). Reverse transcription of total RNA and qPCR with rigorous standard curves were used to measure expression of 16 genes (**B**–**Q**) identified as differentially expressed in the RNA-Seq study. Significant differences between oxygen groups or oxygen by age interactions were confirmed for 14 genes (panels **B** through **O**), but not for 2 genes (panels **P** and **Q**). Expression levels are least squares means (± 1 SE) for each oxygen group at 7 and 9 months of age

Hierarchical clustering of these genes by expression pattern showed separation of normal-sized from enlarged hearts (i.e., the two deepest branches in the dendrogram in Fig. 3A). There was also separation between younger and older turtles (the next deepest branches in the dendrogram). Finally, two distinct clusters contained the N21 and H10 groups from 7-month-old turtles (separation of red and blue branches in top half of the dendrogram). Overall, this pattern of clustering reflected clear expression differences between N21 and H10 groups.

**Fig. 4:**
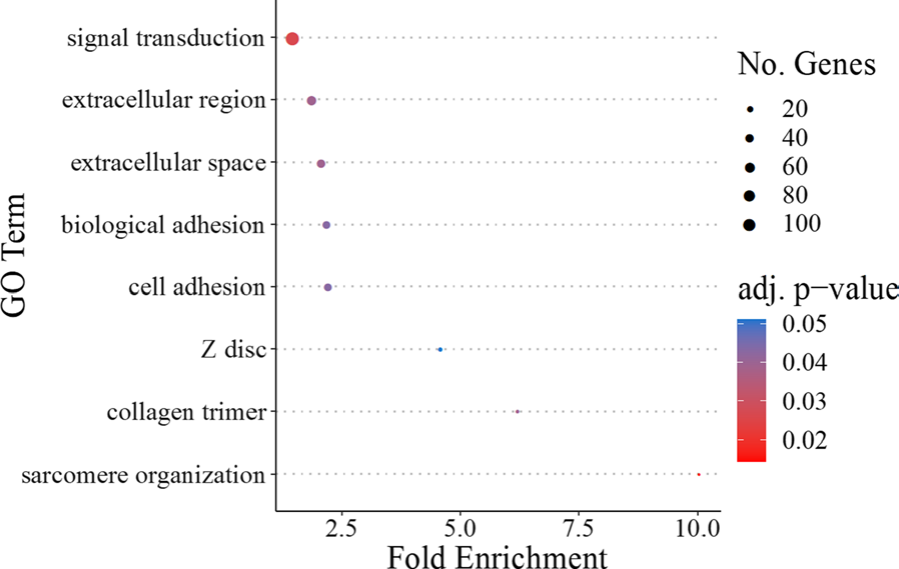
Gene Ontology terms significantly enriched among genes that were differentially expressed in ventricles from juvenile snapping turtles that had been incubated in normoxic or hypoxic conditions as embryos. Enrichment analysis was carried out on all 443 genes that were affected by oxygen concentration during embryogenesis (Table 2), the oxygen concentration by age interaction (Table 3), and/or those genes that differed between ventricles from turtles that had normal-sized vs. enlarged hearts relative to their body size (Table 4). The number of genes for each GO term is represented by the size of the circle, while the FDR adjusted *p* value is shown by the color of the circle

Differentially expressed genes were enriched for several GO terms important for cardiac function and/or remodeling (Fig. 4). For GO Biological Processes, this included 8 differentially expressed genes that play a role in sarcomere organization, 33 genes that play a role in biological adhesion/cell adhesion, and 118 genes involved in signal transduction (Fig. 4; Table 5). For GO Cellular Components, 9 differentially expressed genes form collagen trimers, 11 genes are part of the Z-disc, 41 genes are found in the extracellular space, and 50 genes are part of the extracellular region (Fig. 4; Table 6). Several genes across different GO categories are candidates that might play a role in promoting cardiac anoxia tolerance in the H10 group.

**Table 5:** GO biological process terms and genes that were enriched among differentially expressed genes in ventricles from juvenile snapping turtles

locus_number (in snapping turtle)	gene_name	gene_symbol
Sarcomere organization		
CS000009788	Bone morphogenetic protein 10	BMP10
CS000018274	Cysteine and glycine rich protein 1	CSRP1
CS000002632	Cysteine and glycine rich protein 3	CSRP3
CS000023885	Formin homology 2 domain containing 3	FHOD3
CS000019220	Keratin 8	KRT8
CS000005252	Myomesin 3	MYOM3
CS000000499	Myopalladin	MYPN
CS000000944	Troponin T2, cardiac type	TNNT2
Cell adhesion = biological adhesion		
CS000007175	Adhesion G protein-coupled receptor L3	ADGRL3
CS000009633	Activated leukocyte cell adhesion molecule	ALCAM
CS000004780	Amyloid beta precursor protein-binding family A member 1	APBA1
CS000009788	Bone morphogenetic protein 10	BMP10
CS000003073	Cadherin 11, type 2, OB-cadherin (osteoblast)	CDH11
CS000003093	Cadherin 13	CDH13
CS000014745	Cadherin EGF LAG seven-pass G-type receptor 3	celsr3
CS000008605	Contactin associated protein 1	CNTNAP1
CS000002886	Collagen type XV alpha 1 chain	COL15A1
CS000014058	Collagen, type XVIII, alpha 1	COL18A1
CS000004853	Collagen type III alpha 1 chain	COL3A1
CS000025399	Collagen type IV alpha 6 chain	COL4A6
CS000009656	Collagen type VIII alpha 1 chain	COL8A1
CS000018274	Cysteine and glycine rich protein 1	CSRP1
CS000007341	Catenin delta 1	CTNND1
CS000021606	Ependymin related 1	EPDR1
CS000003405	Fibronectin 1	FN1
CS000001877	Glycoprotein nmb	GPNMB
CS000018642	Hyaluronan-binding protein 2	HABP2
CS000007378	Heat shock protein family B (small) member 1	HSPB1
CS000012953	Immunoglobulin superfamily containing leucine-rich repeat	ISLR
CS000008411	Integrin subunit alpha 11	ITGA11
CS000009567	Keratin 18	KRT18
CS000017005	Muskelin 1	MKLN1
CS000020271	Myosin, light chain 12A, regulatory, non-sarcomeric	MYL12A
CS000000499	myopalladin	MYPN
CS000008885	Neural precursor cell expressed, developmentally down-regulated 9	NEDD9
CS000018597	Neurexin 2	NRXN2
CS000002394	Neurotrimin	NTM
CS000019311	Protein kinase C alpha	PRKCA
CS000011090	Secreted phosphoprotein 1	SPP1
CS000020166	Tweety family member 1	TTYH1
CS000020993	TYRO3 protein tyrosine kinase	TYRO3
Signal transduction		
CS000017075	Adhesion G protein-coupled receptor D2	ADGRD2
CS000009646	Adhesion G protein-coupled receptor G7	ADGRG7
CS000007175	Adhesion G protein-coupled receptor L3	ADGRL3
CS000003393	ArfGAP with GTPase domain, ankyrin repeat and PH domain 1	AGAP1
CS000009633	Activated leukocyte cell adhesion molecule	ALCAM
CS000011198	Adaptor protein, phosphotyrosine interacting with PH domain and leucine zipper 1	APPL1
CS000002385	Rho GTPase-activating protein 32	ARHGAP32
CS000021833	Rho GTPase activating protein 45	ARHGAP45
CS000023302	Cdc42 guanine nucleotide exchange factor 9	ARHGEF9
CS000003395	Ankyrin repeat and SOCS box containing 18	ASB18
CS000001723	ATM serine/threonine kinase	ATM
CS000013883	BCL tumor suppressor 7B	BCL7B
CS000009788	Bone morphogenetic protein 10	BMP10
CS000004917	Bone morphogenetic protein receptor type 2	BMPR2
CS000014425	B-Raf proto-oncogene, serine/threonine kinase	BRAF
CS000009112	Calcium-binding protein 5	CABP5
CS000023033	Calcitonin receptor	CALCR
CS000004923	CD28 molecule	CD28
CS000003093	Cadherin 13	CDH13
CS000014745	Cadherin EGF LAG seven-pass G-type receptor 3	celsr3
CS000006114	chordin like 1	CHRDL1
CS000008605	Contactin associated protein 1	CNTNAP1
CS000002886	Collagen type XV alpha 1 chain	COL15A1
CS000004853	Collagen type III alpha 1 chain	COL3A1
CS000025399	Collagen type IV alpha 6 chain	COL4A6
CS000009979	Cysteine rich transmembrane BMP regulator 1	CRIM1
CS000011960	Crystallin alpha B	CRYAB
CS000002632	Cysteine and glycine rich protein 3	CSRP3
CS000007341	Catenin delta 1	CTNND1
CS000003128	Cytochrome b-245 alpha chain	CYBA
CS000005867	Epidermal growth factor receptor pathway substrate 8	EPS8
CS000008545	Erb-b2 receptor tyrosine kinase 2	ERBB2
CS000012011	Coagulation factor III, tissue factor	F3
CS000002168	Fibroblast growth factor receptor 3	FGFR3
CS000003405	Fibronectin 1	FN1
CS000002902	Gamma-aminobutyric acid type B receptor subunit 1	GABBR1
CS000008168	Glucagon	GCG
CS000005687	G protein subunit alpha 12	GNA12
CS000013318	G protein subunit gamma 10	GNG10
CS000001877	Glycoprotein nmb	GPNMB
CS000017383	G protein-coupled receptor 62	GPR62
CS000008476	Growth factor receptor bound protein 10	GRB10
CS000008544	Growth factor receptor bound protein 7	GRB7
CS000006655	G protein-coupled receptor kinase 3	GRK3
CS000020555	Granzyme H	GZMH
CS000023516	Hypoxia inducible factor 1 alpha subunit	HIF1A
CS000007378	Heat shock protein family B (small) member 1	HSPB1
CS000000714	Interleukin 22	IL22
CS000008411	Integrin subunit alpha 11	ITGA11
CS000005400	Inositol-trisphosphate 3-kinase A	ITPKA
CS000009858	Arginine demethylase and lysine hydroxylase	JMJD6
CS000020495	Kinase non-catalytic C-lobe domain containing 1	KNDC1
CS000009567	Keratin 18	KRT18
CS000019220	Keratin 8	KRT8
CS000015494	Limb development membrane protein 1	LMBR1
CS000008774	Mitogen-activated protein kinase kinase kinase 5	MAP3K5
CS000003679	Mitogen-activated protein kinase 9	MAPK9
CS000001243	Myelin basic protein	MBP
CS000023152	Mindbomb E3 ubiquitin protein ligase 1	MIB1
CS000017005	Muskelin 1	MKLN1
CS000014226	MOB kinase activator 1B	MOB1B
CS000012971	Neuron derived neurotrophic factor	NDNF
CS000008885	Neural precursor cell expressed, developmentally down-regulated 9	NEDD9
CS000001770	Neuroepithelial cell transforming 1	NET1
CS000000790	NFKB inhibitor like 1	NFKBIL1
CS000018597	Neurexin 2	NRXN2
CS000014447	PDZ-binding kinase	PBK
CS000003989	Pleckstrin homology domain interacting protein	PHIP
CS000000947	Pleckstrin homology-like domain family A member 3	PHLDA3
CS000010015	Proteolipid protein 1	PLP1
CS000000163	DNA polymerase kappa	POLK
CS000025418	Peptidylprolyl isomerase A	PPIA
CS000008337	Protein phosphatase, Mg^2+^/Mn^2+^-dependent 1L	PPM1L
CS000000189	Protein kinase AMP-activated non-catalytic subunit beta 2	PRKAB2
CS000019311	Protein kinase C alpha	PRKCA
CS000012528	Persephin	PSPN
CS000009267	RAB32, member RAS oncogene family	RAB32
CS000004444	RAB33A, member RAS oncogene family	RAB33A
CS000007756	Raf-1 proto-oncogene, serine/threonine kinase	RAF1
CS000001871	Rap guanine nucleotide exchange factor 5	RAPGEF5
CS000015645	Regulator of cell cycle	RGCC
CS000020112	Ral guanine nucleotide dissociation stimulator-like 2	RGL2
CS000023359	Ral guanine nucleotide dissociation stimulator like 3	RGL3
CS000008982	Regulator of G-protein signaling 5	RGS5
CS000009817	Rho related BTB domain containing 2	RHOBTB2
CS000011866	Ras homolog family member C	RHOC
CS000000565	Ras and Rab interactor-like protein	RINL
CS000012325	Receptor interacting serine/threonine kinase 2	RIPK2
CS000012890	Sestrin 3	SESN3
CS000021605	Secreted frizzled related protein 4	SFRP4
CS000018595	Signal-induced proliferation-associated 1	SIPA1
CS000005505	SIVA1 apoptosis inducing factor	SIVA1
CS000012858	Suppressor of cytokine signaling 2	SOCS2
CS000023280	Sorbin and SH3 domain containing 2	SORBS2
CS000006802	Sortilin related VPS10 domain containing receptor 1	SORCS1
CS000020596	Sortilin related VPS10 domain containing receptor 2	SORCS2
CS000011090	Secreted phosphoprotein 1	SPP1
CS000021122	Sprouty related EVH1 domain containing 2	SPRED2
CS000003610	Serine/threonine kinase 32A	STK32A
CS000024894	Serine/threonine kinase 38 like	STK38L
CS000024997	Toll like receptor 6	TLR6
CS000015148	Tumor necrosis factor superfamily member 10	TNFSF10
CS000011024	Target of myb1 like 1 membrane trafficking protein	TOM1L1
CS000019856	TNF receptor associated factor 2	TRAF2
CS000012224	TNF receptor associated factor 3	TRAF3
CS000018042	Tribbles pseudokinase 1	TRIB1
CS000002912	Tripartite motif containing 21	TRIM21
CS000005987	Tyrosine kinase 2	TYK2
CS000020993	TYRO3 protein tyrosine kinase	TYRO3
CS000017504	unc-5 netrin receptor A	UNC5A
CS000002566	VAMP associated protein B and C	VAPB
CS000025210	Wnt family member 11	WNT11
CS000014907	Zinc finger protein 219	ZNF219
CS000002650	Doublecortin domain containing 1	
CS000013923	Uncharacterized	
CS000014046	G protein-coupled receptor kinase 5-like	
CS000019987	Plexin A3	
CS000025011	Retinoic acid receptor, alpha	

Gene names and gene symbols are listed for each enriched GO term. Genes in the biological adhesion and cell adhesion lists were identical, because these are parent and child terms. GO terms were considered significant when FDR corrected *p* ≤ 0.05

**Table 6:** GO Cellular Component terms and genes that were enriched among differentially expressed genes in ventricles from juvenile snapping turtles

locus_number (in snapping turtle)	gene_name	gene_symbol
Collagen trimer		
CS000004853	Collagen type III alpha 1 chain	COL3A1
CS000025399	Collagen type IV alpha 6 chain	COL4A6
CS000009656	Collagen type VIII alpha 1 chain	COL8A1
CS000002886	Collagen type XV alpha 1 chain	COL15A1
CS000014058	Collagen, type XVIII, alpha 1	COL18A1
CS000002530	Collagen type XX alpha 1 chain	COL20A1
CS000018990	Collagen type XXII alpha 1 chain	COL22A1
CS000007237	Collectin subfamily member 10	COLEC10
CS000014966	Ficolin 3	FCN3
z-disc		
CS000009788	Bone morphogenetic protein 10	BMP10
CS000011960	Crystallin alpha B	CRYAB
CS000018274	Cysteine and glycine rich protein 1	CSRP1
CS000002632	Cysteine and glycine rich protein 3	CSRP3
CS000023885	Formin homology 2 domain containing 3	FHOD3
CS000007378	Heat shock protein family B (small) member 1	HSPB1
CS000019220	Keratin 8	KRT8
CS000005252	Myomesin 3	MYOM3
CS000000499	Myopalladin	MYPN
CS000015509	Sodium voltage-gated channel alpha subunit 5	SCN5A
CS000023280	Sorbin and SH3 domain containing 2	SORBS2
Extracellular space		
CS000008845	Apolipoprotein F	APOF
CS000009788	Bone morphogenetic protein 10	BMP10
CS000004917	Bone morphogenetic protein receptor type 2	BMPR2
CS000004554	Complement C1r	C1R
CS000021043	Complement C1s	C1S
CS000023075	CD109 molecule	CD109
CS000003093	Cadherin 13	CDH13
CS000003059	Carboxylesterase 2	CES2
CS000010784	Cholesteryl ester transfer protein	CETP
CS000014980	Complement factor B	CFB
CS000008574	2′,3′-Cyclic nucleotide 3′ phosphodiesterase	CNP
CS000002886	Collagen type XV alpha 1 chain	COL15A1
CS000014058	Collagen, type XVIII, alpha 1	COL18A1
CS000002530	Collagen type XX alpha 1 chain	COL20A1
CS000018990	Collagen type XXII alpha 1 chain	COL22A1
CS000004853	Collagen type III alpha 1 chain	COL3A1
CS000025399	Collagen type IV alpha 6 chain	COL4A6
CS000009656	Collagen type VIII alpha 1 chain	COL8A1
CS000007237	Collectin subfamily member 10	COLEC10
CS000005701	Extracellular leucine rich repeat and fibronectin type III domain containing 1	ELFN1
CS000012011	Coagulation factor III, tissue factor	F3
CS000003405	Fibronectin 1	FN1
CS000008168	Glucagon	GCG
CS000018642	Hyaluronan-binding protein 2	HABP2
CS000007378	Heat shock protein family B (small) member 1	HSPB1
CS000000714	Interleukin 22	IL22
CS000005377	Galectin 1	LGALS1
CS000024186	Leucine rich repeat containing 3B	LRRC3B
CS000000330	Matrix metallopeptidase 25	MMP25
CS000001051	Monooxygenase DBH like 1	MOXD1
CS000025418	Peptidylprolyl isomerase A	PPIA
CS000007586	Proline and arginine rich end leucine rich repeat protein	PRELP
CS000012528	Persephin	PSPN
CS000006963	Secretogranin II	SCG2
CS000024555	Stimulator of chondrogenesis 1	SCRG1
CS000021605	Secreted frizzled related protein 4	SFRP4
CS000008622	Sclerostin	sost
CS000011090	Secreted phosphoprotein 1	SPP1
CS000002388	Suppression of tumorigenicity 14	ST14
CS000025210	Wnt family member 11	WNT11
CS000012404	Uncharacterized	
CS000021864	PZP, alpha-2-macroglobulin like	
Extracellular region		
CS000014235	Anthrax toxin receptor 2	ANTXR2
CS000003440	Apolipoprotein C1	apoc1
CS000021833	Rho GTPase activating protein 45	ARHGAP45
CS000009788	Bone morphogenetic protein 10	BMP10
CS000004554	Complement C1r	C1R
CS000021043	Complement C1s	C1S
CS000010941	Coiled-coil domain containing 40	CCDC40
CS000023075	CD109 molecule	CD109
CS000003093	Cadherin 13	CDH13
CS000010784	Cholesteryl ester transfer protein	CETP
CS000014980	Complement factor B	CFB
CS000006114	Chordin like 1	CHRDL1
CS000002886	Collagen type XV alpha 1 chain	COL15A1
CS000014058	Collagen, type XVIII, alpha 1	COL18A1
CS000002530	Collagen type XX alpha 1 chain	COL20A1
CS000018990	Collagen type XXII alpha 1 chain	COL22A1
CS000004853	Collagen type III alpha 1 chain	COL3A1
CS000025399	Collagen type IV alpha 6 chain	COL4A6
CS000009656	Collagen type VIII alpha 1 chain	COL8A1
CS000007237	Collectin subfamily member 10	COLEC10
CS000009979	Cysteine rich transmembrane BMP regulator 1	CRIM1
CS000011191	Deoxyribonuclease 1 like 3	DNASE1L3
CS000021606	Ependymin related 1	EPDR1
CS000013674	Eva-1 homolog C	EVA1C
CS000014966	Ficolin 3	FCN3
CS000002168	Fibroblast growth factor receptor 3	FGFR3
CS000003405	Fibronectin 1	FN1
CS000002902	Gamma-aminobutyric acid type B receptor subunit 1	GABBR1
CS000008168	Glucagon	GCG
CS000009498	Glypican 3	GPC3
CS000018642	Hyaluronan-binding protein 2	HABP2
CS000000714	Interleukin 22	IL22
CS000012953	Immunoglobulin superfamily containing leucine-rich repeat	ISLR
CS000005377	Galectin 1	LGALS1
CS000012971	Neuron derived neurotrophic factor	NDNF
CS000021269	Neural EGFL like 1	NELL1
CS000012602	Neuritin 1	NRN1
CS000002394	Neurotrimin	NTM
CS000025418	Peptidylprolyl isomerase A	PPIA
CS000007586	Proline and arginine rich end leucine rich repeat protein	PRELP
CS000012962	Protease, serine 12	PRSS12
CS000012528	Persephin	PSPN
CS000013172	Sperm flagellar 2	SPEF2
CS000011090	Secreted phosphoprotein 1	SPP1
CS000019009	Thrombospondin type 1 domain containing 7A	THSD7A
CS000015148	Tumor necrosis factor superfamily member 10	TNFSF10
CS000011540	von Willebrand factor A domain containing 3A	VWA3A
CS000013624	von Willebrand factor A domain containing 5B1	VWA5B1
CS000025210	Wnt family member 11	WNT11
CS000012404	Uncharacterized	

Gene names and gene symbols are listed for each enriched GO term. GO terms were considered significant when FDR corrected *p* ≤ 0.05

We selected genes for qPCR validation from the GO categories described above based on their established role in influencing cardiac function and anoxia tolerance, including genes associated with heart defects in humans or other species, genes involved in calcium signaling or mitochondrial function, and/or genes that regulate expression of other genes. Overall, differential expression was confirmed for 14 of 16 genes examined (Table 7; Fig. 3B–Q). Some genes, such as *DDIT4L* and *WNT11*, were expressed at consistently lower levels in the H10 group compared to the N21 group at both ages (Fig. 3B, C). Other genes, such as *ITGA11*, *MIPEP*, *MNAT1*, *PPIA*, *TNNT2*, and *TPM3*, were reliably higher in the H10 group compared to the N21 group (Figs. 3D–I). Several genes displayed treatment by age interactions. For *COL8A1*, *NCOA2, * and *SCN5A* there was no difference at 7 months of age, but expression was higher in the H10 group than the N21 group at 9 months of age (Fig. 3J–L). For *HIF1A* and *PFKFB1*, there was no difference at 7 months of age, but expression was lower in the H10 group than the N21 group at 9 months of age (Fig. 3M, N). Another pattern was observed for *HTRA3*, which differed between treatment groups at 7 months of age, but not at 9 months of age (Fig. 3O). In contrast, *CALR* and *SNTB1* did not differ between N21 and H10 groups at either age (Fig. 3P, Q).

**Table 7:** Results from a two-way ANCOVA for mRNA expression in ventricles from juvenile snapping turtles

Gene	Oxygen treatment	Age	Oxygen treatment × age	Covariate
CALR	*F*_1,20_ = 0.27, *p* = 0.61	*F*_1,20_ = 4.5, ***p* = *0.05***	*F*_1,20_ = 0.18, *p* = 0.68	*F*_1,20_ = 0.71, *p* = 0.41
COL8A1	*F*_1,20_ = 6.9, ***p* = *0.02***	*F*_1,20_ = 15.5, ***p* = *0.0008***	*F*_1,20_ = 3.6, *p* = 0.07	*F*_1,20_ = 8.3, ***p* = *0.009***
DDIT4L	*F*_1,20_ = 9.3, ***p* = *0.006***	F_1,20_ = 0.21, *p* = 0.65	*F*_1,20_ = 0.20, *p* = 0.66	*F*_1,20_ = 14.4, ***p* = *0.001***
HIF1A	*F*_1,20_ = 3.85, *p* = 0.06	*F*_1,20_ = 0.04, *p* = 0.85	*F*_1,20_ = 6.0, ***p* = *0.02***	*F*_1,20_ = 0.96, *p* = 0.34
HTRA3	*F*_1,19_ = 5.2, ***p* = *0.03***	*F*_1,19_ = 3.9, *p* = 0.06	*F*_1,19_ = 6.2, ***p* = *0.02***	*F*_1,19_ = 6.0, ***p* = *0.02***
ITGA11	*F*_1,20_ = 5.4, ***p* = *0.03***	*F*_1,20_ = 4.4, ***p* = *0.05***	*F*_1,20_ = 0.66, *p* = 0.43	*F*_1,20_ = 0.95, *p* = 0.34
MIPEP	*F*_1,20_ = 8.2, ***p* = *0.01***	*F*_*1*,20_ = 0.14, *p* = 0.71	*F*_1,20_ = 0.04, *p* = 0.85	*F*_1,20_ = 8.9, ***p* = *0.007***
MNAT1	*F*_1,20_ = 9.2, ***p* = *0.006***	*F*_1,20_ = 3.1, *p* = 0.09	*F*_1,20_ = 0.48, *p* = 0.5	*F*_1,20_ = 0.08, *p* = 0.78
NCOA2	*F*_1,20_ = 5.9, ***p* = *0.02***	*F*_1,20_ = 5.1, ***p* = *0.03***	*F*_1,20_ = 2.26, *p* = 0.15	*F*_1,20_ = 7.9, ***p* = *0.01***
PFKFB1	*F*_1,20_ = 20.8, ***p* = *0.0002***	*F*_1,20_ = 10.4, ***p* = *0.004***	*F*_1,20_ = 15.4, ***p* = *0.0008***	*F*_1,20_ = 2.2, *p* = 0.15
PPIA	*F*_1,20_ = 5.3, ***p* = *0.03***	*F*_1,20_ = 3.3, *p* = 0.08	*F*_1,20_ = 0.24, *p* = 0.63	*F*_1,20_ = 0.17, *p* = 0.69
SCN5A	*F*_1,17_ = 4.6, ***p* = *0.05***	*F*_1,17_ = 0.04, *p* = 0.85	*F*_1,17_ = 7.3, ***p* = *0.015***	*F*_1,17_ = 16.5, ***p* = *0.0008***
SNTB1	*F*_1,20_ = 0.05, *p* = 0.82	*F*_1,20_ = 4.3, ***p* = *0.05***	*F*_1,20_ = 0.79, *p* = 0.38	*F*_1,20_ = 0.003, *p* = 0.96
TNNT2	*F*_1,20_ = 6.0, ***p* = *0.02***	*F*_1,20_ = 0.01, *p* = 0.93	*F*_1,20_ = 0.13, *p* = 0.72	*F*_1,20_ = 0.12, *p* = 0.74
TPM3	*F*_1,20_ = 12.5, ***p* = *0.002***	*F*_1,20_ = 1.6, *p* = 0.22	*F*_1,20_ = 0.32, *p* = 0.58	*F*_1,20_ = 1.8, *p* = 0.19
WNT11	*F*_1,20_ = 4.6, ***p* = *0.04***	*F*_1,20_ = 3.4, *p* = 0.08	*F*_1,20_ = 0.01, *p* = 0.92	*F*_1,20_ = 1.3, *p* = 0.27

Oxygen treatment, age, and the oxygen treatment by age interaction were fixed effects in the model. The first principal component from a principal components analysis of *CACNA2D1*, *CNP*, and *YTHDF3* expression was used as the covariate in the ANCOVA. Significant effects (*p* ≤ 0.05) are highlighted in bolditalics

### Genome-wide correlation between DNA methylation and gene expression

**Fig. 5:**
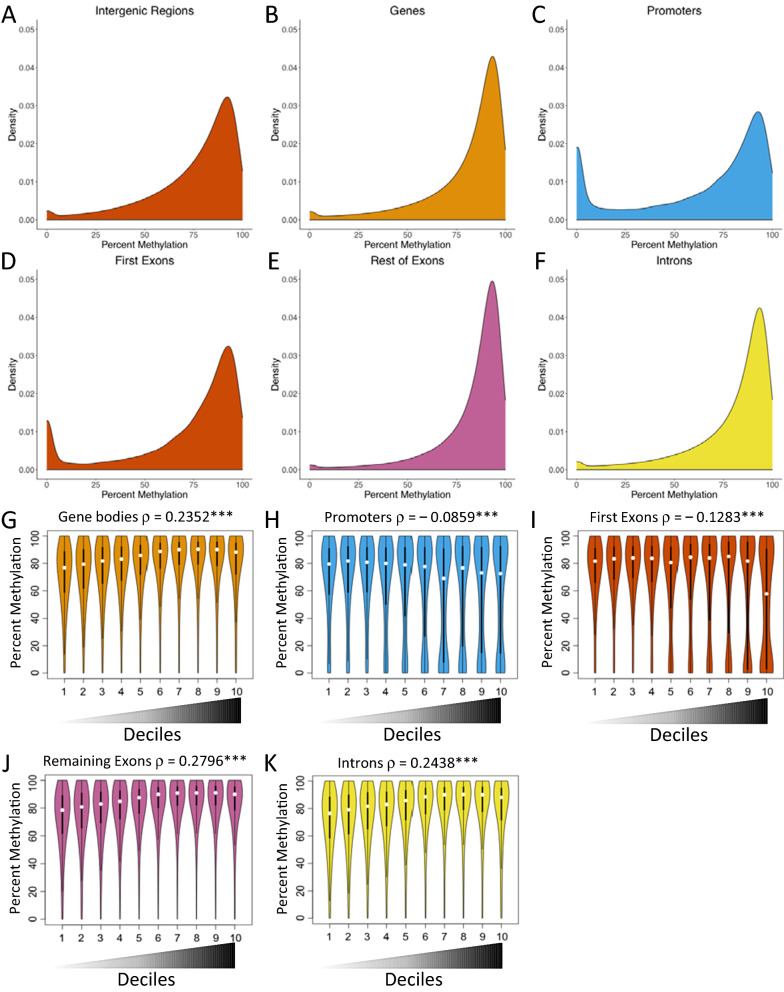
CpG methylation and gene expression patterns in the ventricles of juvenile snapping turtles. Probability density function for CpG methylation level in various genome features (**A**–**F**). **A** Intergenic regions (*n* = 12,840,311), **B** gene bodies (*n* = 3,426,421), **C** promoters (*n* = 68,168), **D** first exons (*n* = 40,226), **E** remaining exons (242,295), and **F** introns (*n* = 3,1561,179). Violin plots showing CpG methylation levels for various gene features as a function of gene expression level (**G**–**K**). Genes were divided into expression deciles with the lowest expressed genes in the first decile and the highest expressed genes in the tenth decile as shown on the *X* axis. The width of the violin corresponds to the probability density function for the methylation level shown on the *Y* axis. The bar in the middle of each violin shows the interquartile range and the white dot shows the median methylation level for each expression decile. Spearman’s rank correlations (*ρ*) between DNA methylation and gene expression were highly significant for all gene features (***p < 0.001). **G** Gene bodies (*n* = 3,426,421), **H** promoters (*n* = 68,168), **I** first exons (*n* = 40,226), **J** remaining exons (242,295), and **K** introns (*n* = 3,1561,179)

DNA samples from ventricles of 9-month-old turtles were used for WGBS (*n* = 3 from N21 and *n* = 3 from H10). Given that DNA methylation landscapes have never been examined at a genome-wide scale in any reptile, basic patterns of DNA methylation were characterized before testing for differences between treatment groups. The draft snapping turtle genome contains approximately 142.4 million CpG dinucleotides. The distribution of CGIs in different genomic features was not random with respect to the proportion of the genome found in promoters, gene bodies, and intergenic regions: more CGIs were found in promoters (0 to − 1000 bp from the transcription start site) and in gene bodies than expected by chance, while fewer CGIs were found in intergenic regions (Table 8). For CpGs with sufficient read coverage (≥ 10 reads in 2/3 of replicates), levels of methylation were high (two thirds of CpGs across the genome were 75–100% methylated) and there were clear differences in methylation patterns among genomic features (Fig. 5). Intergenic regions (Fig. 5A) had a broader range of DNA methylation levels than did gene bodies (Fig. 5B). Intergenic regions had a higher proportion of CpGs with 0–75% methylation and a lower proportion of CpGs with 75–100% methylation than did gene bodies (Table 9). In other words, gene bodies were more heavily methylated than intergenic regions.

**Table 8:** Summary of the observed and the expected number of CGIs in different genomic features

Genome feature	Length of feature in genome	Proportion of genome	Observed CpG Islands	Expected CpG Islands
Intergenic region	2,167,077,285	0.96	109,766	193,725
Promoter	22,812,000	0.01	4689	2039
Gene body	67,834,108	0.03	87,373	6064
Total	2,257,723,393	1.0	201,828	201,828

Expected numbers of CGIs in intergenic regions, promoters, and gene bodies were calculated based on the proportion of the snapping turtle genome found in each of these genomic features

**Table 9:** Summary of the number of CpG sites by their genomic location and methylation level

Genome feature	0–25% Methylation	25–50% Methylation	50–75% Methylation	75–100% Methylation	Total CpGs
Intergenic region	589,261 (4.6%)	1,149,789 (8.9%)	3,112,308 (24.2%)	7,988,953 (62.2%)	12,840,311
Gene body	132,403 (3.9%)	212,133 (6.2%)	607,207 (17.7%)	2,474,678 (72.2%)	3,426,421
Promoter	13,500 (19.8%)	5950 (8.7%)	12,777 (18.7%)	35,941 (52.7%)	68,168
First exon	5000 (12.4%)	2755 (6.8%)	7914 (19.7%)	24,557 (61.0%)	40,226
Remaining exons	5649 (2.3%)	10,047 (4.1%)	35,845 (14.8%)	190,754 (78.7%)	242,295
Introns	122,441 (3.9%)	199,976 (6.3%)	566,193 (17.9%)	2,267,569 (71.8%)	3,156,179

Sites are binned into four levels of methylation: 0–25%, 25–50%, 50–75%, and 75–100%

There were also differences in CpG methylation patterns among gene features. Promoters (0 to − 1000 bp from the transcription start site or TSS) and first exons displayed a bimodal pattern of DNA methylation (Fig. 5C, D; Table 9), with a higher proportion of CpGs with 0–25% methylation (including unmethylated CpGs) and a lower proportion of CpGs with 75–100% methylation when compared to gene bodies, remaining exons, and introns (Fig. 5B, E, F; Table 9). That is, promoters and first exons displayed more variation in methylation levels and were less methylated on average than other exons and introns.

To test whether there was any relationship between CpG methylation and gene expression, genes expressed at a detectable level in turtle ventricles were divided into deciles based on expression levels with the first decile containing genes that displayed the lowest expression and the tenth decile containing genes that displayed the highest expression. There was a positive correlation between CpG methylation in gene bodies and expression levels (Fig. 5G). In contrast, CpG methylation in promoters and first exons was negatively correlated with gene expression (Fig. 5H, I). Remaining exons and introns displayed a positive correlation between CpG methylation and gene expression (Fig. 5J, K).

**Fig. 6:**
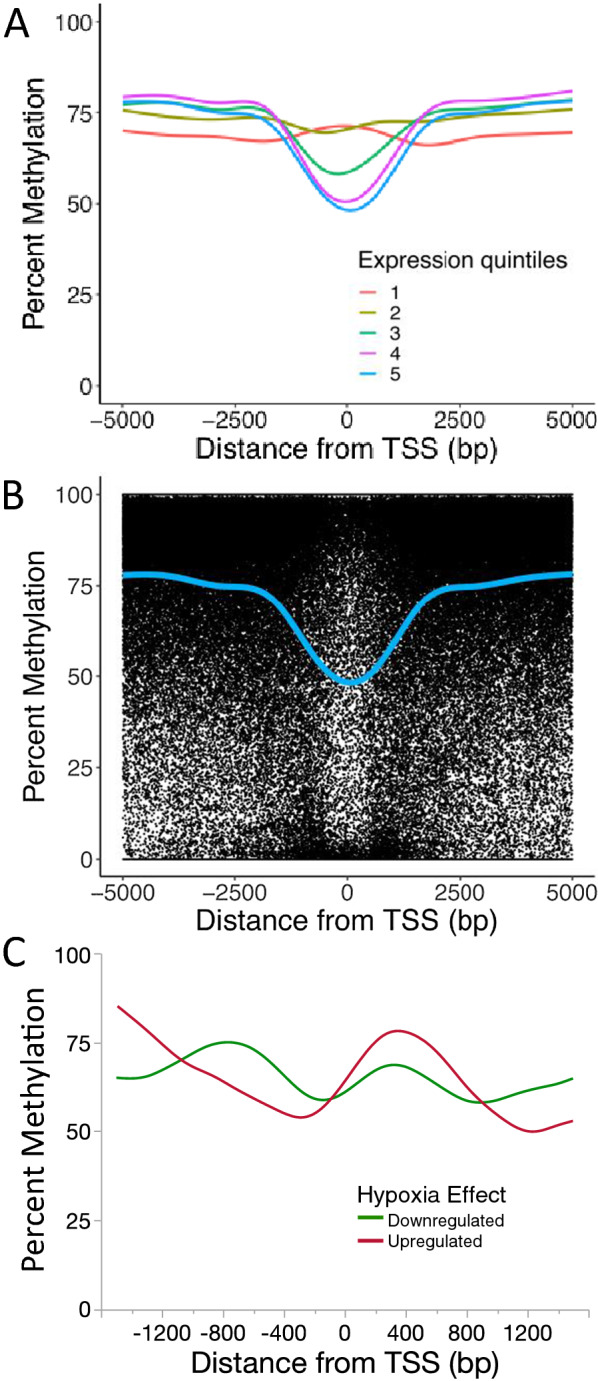
CpG methylation levels around TSSs as a function of gene expression level in ventricles of juvenile snapping turtles. **A** Genes were divided into expression quintiles with the lowest expressed genes in the first quintile and the highest expressed genes in the fifth quintile. Methylation level for each quintile was plotted as a function of the distance from the TSS. Lines were produced via LOESS curve fitting. **B** Scatter plot showing methylation level for each CpG as a function distance from the TSS for genes in the fifth quintile. The blue line is the same LOESS smoothed curve as shown in the previous panel. **C** CpG methylation level around TSSs for genes that were differentially expressed in ventricles of 9-month-old snapping turtles that had been incubated in normoxic (N21) or hypoxic (H10) conditions as embryos. Methylation levels are shown for genes that were up-regulated (red line) or down-regulated (green line) in hypoxic turtles compared to normoxic turtles

CpG methylation levels were plotted as a function of distance from TSSs to examine the methylation landscape of promoters at a finer spatial scale. Genes were divided into quintiles based on expression levels with the first quintile containing genes with the lowest expression and the fifth quintile containing genes with the highest expression. There was a clear negative correlation between methylation and gene expression levels (Fig. 6A). Genes in the first expression quintile exhibited slightly higher CpG methylation near the TSS vs. neighboring sites (i.e., a hill). In contrast, genes in the second through fifth expression quintiles exhibited progressively lower CpG methylation near the TSS (i.e., greater depth of the methylation valley with increasing expression). This valley spanned from roughly 1500 bp upstream to 1500 bp downstream of the TSS (Fig. 6A). A scatterplot of methylation levels for individual CpGs for genes in the fifth quintile showed a clear bimodal pattern centered on the TSS (i.e., most sites displaying 0% or 100% methylation) (Fig. 6B). This analysis demonstrated an inverse relationship between CpG methylation and gene expression: higher methylation at TSSs was associated with lower gene expression, while lower methylation at TSSs was associated with higher expression at a genome-wide scale.

### Embryonic hypoxia programs genome-wide patterns of CpG and CpG island methylation

Given that CpG methylation patterns were broadly correlated with gene expression in turtle hearts, fetal programming of DNA methylation could be driving hypoxia-induced differences in gene expression and physiology. The first step toward testing this hypothesis is to determine whether embryonic exposure to hypoxia caused differential DNA methylation. CpGs and CGIs were examined separately, because methylation patterns in mammals differ between isolated CpGs (heavily methylated) vs. CGIs (lightly methylated), as does the relationship of CpG and CGI methylation to gene expression.

Comparison of N21 and H10 groups revealed 74,016 differentially methylated CpGs out of 10,808,104 CpGs with sufficient coverage for analysis and difference > 25% and *q* < 0.01. Hypoxic conditions during embryogenesis induced hypermethylation of 38,428 CpGs and hypomethylation of 35,588 CpGs. Intergenic regions were not more or less likely to contain differentially methylated CpGs than expected by chance (Odds Ratio = 1.014, 95% CI = 0.999 to 1.029; Fisher’s Exact *p* = 0.066). However, differentially methylated CpGs were more likely to be found in promoters than expected by chance (Odds Ratio = 1.148, 95% CI = 1.057 to 1.245; Fisher’s Exact *p* = 0.001). In contrast, differentially methylated CpGs were less likely to be found in first exons (Odds Ratio = 0.707, 95% CI = 0.613 to 0.811; Fisher’s Exact *p* = 2e−07) or the remaining exons (Odds Ratio = 0.510, 95% CI = 0.481 to 0.541; Fisher’s Exact *p* = 2e−16).

Comparison of N21 and H10 groups revealed 6,666 differentially methylated CGIs (FDR < 0.05). Hypoxic conditions during embryogenesis induced hypermethylation of 3628 CGIs and hypomethylation of 3038 CGIs. Intergenic regions were more likely to contain differentially methylated CGIs than expected by chance (Odds Ratio = 1.136, 95% CI = 1.078 to 1.197; Fisher’s Exact *p* = 1.33e−6). In contrast, differentially methylated CGIs were less likely to be found in promoters than expected by chance (Odds Ratio = 0.738, 95% CI = 0.596 to 0.907; Fisher’s Exact *p* = 0.003). Although not statistically significant, a trend toward fewer differentially methylated CGIs was also observed in first exons (Odds Ratio = 0.815, 95% CI = 0.654 to 1.006; Fisher’s Exact *p* = 0.06) and the remaining exons (Odds Ratio = 0.847, 95% CI = 0.702 to 1.016; Fisher’s Exact *p* = 0.07).

### Functional enrichment among differentially methylated genes

**Fig. 7:**
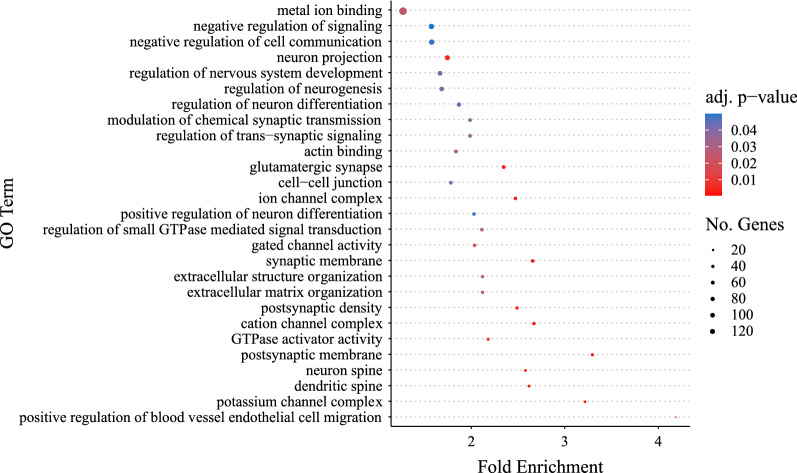
Gene Ontology terms significantly enriched among genes that were differentially methylated in ventricles from juvenile snapping turtles that had been incubated in normoxic or hypoxic conditions as embryos. Enrichment analysis was carried out on 1582 genes that were affected by oxygen concentration during embryogenesis. The number of genes for each GO term is represented by the size of the circle, while the FDR adjusted *p* value is shown by the color of the circle

For GO analysis, differentially methylated genes (*n* = 1582) were defined as those containing ≥ 1 differentially methylated region (methylKit 200 bp sliding window with 50 bp steps) within their promoter (− 1000 bp from TSS) and/or gene body at a *q* < 0.001 (Additional file 1: Table S1). Differentially methylated genes were significantly enriched for numerous GO terms at a Bonferroni corrected *p* < 0.05 (Fig. 7). Eighteen terms were significant for GO Biological Process, including six GO terms that might be related to differences in the autonomic nervous system and bradycardia between N21 and H10 groups (Fig. 7; Additional file 2: Table S2). Among these, the highest level terms include “regulation of trans-synaptic signaling”, “regulation of nervous system development”, and “regulation of neuron differentiation” (Fig. 7; Additional file 2: Table S2). Thirty-one GO terms were significant for GO Cellular Component (Fig. 7; Additional file 2: Table S2). Several of these terms were also related to neuronal function, while other terms were related to cation channels that could play a role in positive ionotropic responses in the H10 group. Finally, GO Molecular Function contained 10 terms that complement GO Biological Process and Cellular Component terms (Fig. 7; Additional file 2: Table S2).

### Correlation between hypoxia-induced DNA methylation and gene expression patterns

Having demonstrated that embryonic exposure to hypoxic conditions programmed differential methylation of CpGs and CGIs in juvenile turtle hearts, it was possible to test for relationships to hypoxia-induced differences in gene expression. Genes containing at least one differentially methylated region (as defined in the previous paragraph) were more likely to be differentially expressed than expected by chance (Odds Ratio = 1.558, 95% CI = 1.178 to 2.059; Fisher’s Exact *p* = 0.002). Genes that were both differentially methylated and differentially expressed between the N21 and H10 groups are listed in Table 10. Given the negative correlation between CpG methylation in promoters and gene expression (Fig. 5H) and the clear methylation signal centered on TSSs (Fig. 6A), finer scale CpG methylation patterns were examined for genes that were differentially expressed between the H10 and N21 groups at 9 months of age. Genes that were up-regulated and down-regulated by hypoxic incubation exhibited spatially distinct methylation patterns, particularly in the − 200 to − 1000 bp region of promoters (Fig. 6C). Although differences were not as stark as observed at a genome-wide scale, there was higher methylation in this region for genes that were downregulated by hypoxic incubation and lower methylation for upregulated genes. Taken together, these findings suggest that hypoxia-induced differences in CpG methylation in promoters and/or gene bodies contribute to hypoxia-induced differences in gene expression.

**Table 10:** Genes that were both differentially methylated and differentially expressed between ventricles from snapping turtles that were exposed to normoxia (N21) or hypoxia (H10) during embryonic development

locus_number	gene_symbol	gene_name
CS000003610	STK32A	Serine/threonine kinase 32A
CS000001243	MBP	Myelin basic protein
CS000013998	PNPLA7	Patatin like phospholipase domain containing 7
CS000014058	COL18A1	Collagen, type XVIII, alpha 1
CS000013081	PRDM8	PR/SET domain 8
CS000013911		
CS000014425	BRAF	B-Raf proto-oncogene, serine/threonine kinase
CS000014649	HN1	Hematological and neurological expressed 1
CS000008916	TMEM214	Transmembrane protein 214
CS000003393	AGAP1	ArfGAP with GTPase domain, ankyrin repeat and PH domain 1
CS000005022	DNAJC11	DnaJ heat shock protein family (Hsp40) member C11
CS000004897	TMEM237	Transmembrane protein 237
CS000002385	ARHGAP32	Rho GTPase-activating protein 32
CS000002394	NTM	Neurotrimin
CS000012858	SOCS2	Suppressor of cytokine signaling 2
CS000008411	ITGA11	Integrin subunit alpha 11
CS000008476	GRB10	Growth factor receptor bound protein 10
CS000013316	KIAA0368	KIAA0368
CS000012086		Actin, aortic smooth muscle-like
CS000012122		Astacin-like metalloendopeptidase
CS000001723	ATM	ATM serine/threonine kinase
CS000008885	NEDD9	Neural precursor cell expressed, developmentally down-regulated 9
CS000018434		
CS000006802	SORCS1	Sortilin related VPS10 domain containing receptor 1
CS000018642	HABP2	Hyaluronan-binding protein 2
CS000006855		Transforming acidic coiled-coil-containing protein 2
CS000011540	VWA3A	von Willebrand factor A domain containing 3A
CS000018526	MOSMO	Modulator of smoothened protein
CS000011558	TMC5	Transmembrane channel like 5
CS000025151	JARID2	Jumonji and AT-rich interaction domain containing 2
CS000019009	THSD7A	Thrombospondin type 1 domain containing 7A
CS000019311	PRKCA	Protein kinase C alpha
CS000003093	CDH13	Cadherin 13
CS000019541		Chromosome 21 orf 58
CS000004780	APBA1	Amyloid beta precursor protein-binding family A member 1
CS000002168	FGFR3	Fibroblast growth factor receptor 3
CS000002632	CSRP3	Cysteine and glycine rich protein 3
CS000002650		Doublecortin domain containing 1
CS000021269	NELL1	Neural EGFL like 1
CS000002886	COL15A1	Collagen type XV alpha 1 chain
CS000009633	ALCAM	Activated leukocyte cell adhesion molecule
CS000009656	COL8A1	Collagen type VIII alpha 1 chain
CS000004132	LDLRAD4	Low density lipoprotein receptor class A domain containing 4
CS000023075	CD109	CD109 molecule
CS000022474		Dedicator of cytokinesis 2
CS000009979	CRIM1	Cysteine rich transmembrane BMP regulator 1
CS000000757	TDRP	Testis development related protein
CS000012971	NDNF	Neuron derived neurotrophic factor
CS000003458	SMARCAD1	SWI/SNF-related, matrix-associated actin-dependent regulator of chromatin, subfamily a, containing DEAD/H box 1
CS000003463	ADH4	Alcohol dehydrogenase 4 (class II), pi polypeptide
CS000003466	TRMT10A	tRNA methyltransferase 10A
CS000004598		
CS000004600		
CS000008823	NCOA2	Nuclear receptor coactivator 2
CS000023885	FHOD3	Formin homology 2 domain containing 3
CS000024095	SNTB1	Syntrophin beta 1
CS000024278	RALYL	RALY RNA-binding protein-like
CS000024331	CERS4	Ceramide synthase 4
CS000012404		

Distal regulatory elements such as enhancers and silencers are another important factor driving gene expression patterns. Recent work in mammals has shown that orphan CGIs can act as enhancers. It is, therefore, possible that differential methylation of “CGI enhancers” could influence gene expression patterns in the snapping turtle. As a preliminary test of this hypothesis, we identified the closest gene to the 6666 differentially methylated CGIs described above. There were 4379 protein-coding genes near these sites. We then tested whether these genes were more or less likely to be differentially expressed between N21 and H10 groups. Genes closest to differentially methylated CGIs were less likely to be differentially expressed than expected by chance (Odds Ratio = 0.732, 95% CI = 0.578 to 0.927; Fisher’s Exact *p* = 0.005). Long-distance enhancer–promoter interactions that skip over the closest gene may explain this result (see discussion for in-depth consideration of this idea).

### Putative *cis*-regulatory sequences for fetal programming of DNA methylation

HOMER2 was used to identify *cis*-regulatory elements that could be directing differential DNA methylation and/or differential gene expression between N21 and H10 groups. Proximal promoters (− 1000 bp to TSS) of the 443 differentially expressed genes were retrieved from the snapping turtle genome and analyzed for known and de novo sequence motifs. Known motifs for androgen response elements and glucocorticoid response elements were found in promoters at a higher frequency than expected by chance, but were not significant after FDR correction. Thirty-three (33) de novo sequence motifs were enriched in promoters (Additional file 3: Table S3). As expected for promoters, HOMER2 identified TATA boxes and downstream core elements (DCEs), which are recognized by TATA-binding protein and TFIID, respectively, at TSSs. The two highest scoring de novo motifs, which exceeded a stringent cutoff of 1e−10, were similar to sequences bound by transcription factors ZNF711 and RREB1. Several de novo sequence motifs are recognized by transcription factors (CEBPB, KLF10, MEIS1, RREB1, and RXRA) known to bind methylated DNA in mammals [29].

Given that CGIs can act as enhancers in other species, it is possible that differential methylation of CGIs could modulate their activity as enhancers in turtles. HOMER2 was, therefore, used to identify motifs within the 6,666 differentially methylated CGIs. A total of 42 known sequence motifs were enriched in differentially methylated CGIs with an FDR < 0.005 (Additional file 4: Table S4). Three transcription factors that bind to these motifs play a central role in mediating the effects of hypoxia on gene expression: i.e., HIF1A, ARNT (encodes Hif1-beta), and EPAS1 (encodes Hif2-alpha)-binding sites were all enriched in differentially methylated CGIs. Several transcription factors (CUX2, GABPA, GSC, PHOX2B, SMAD4, and ZEB2) that bind to enriched sequence motifs are associated with human syndromes that exhibit cardiovascular, mitochondrial, or autonomic nervous system defects (Additional file 4: Table S4). Several transcription factors (CRX, ZBTB33, SMAD4) are also known to bind methylated sites [29].

## Discussion

The present study provides the first ever genome-wide analysis of DNA methylation patterns in a reptile and gives a detailed characterization of the methylation landscape across the snapping turtle genome. In addition, we show that developmental hypoxia is a potent environmental stimulus that alters snapping turtle DNA methylation patterns, which are associated with changes in gene expression and improved performance of the cardiovascular system during anoxia. Surprisingly, juvenile turtles from hypoxic incubations were able to maintain cardiac pumping capacity throughout 2 h of anoxia at 30 °C, which is a feat unsurpassed among vertebrates. To understand the molecular mechanisms underlying programmed differences in cardiac physiology, we carried out WGBS and RNA-Seq studies in ventricular tissue from the H10 and N21 cohorts. Developmental hypoxia programmed genome-wide methylation patterns at CpGs and CGIs. Furthermore, DNA methylation patterns were broadly correlated with gene expression at a genome-wide scale as well as for genes that were differentially expressed between normoxic and hypoxic turtles. Finally, we identified enriched DNA sequence motifs (i.e., putative TF-binding sites) in promoters of differentially expressed genes and in differentially methylated CGIs. By integrating this information, we develop a hypothetical model that links hypoxia during embryogenesis to persistent changes in DNA methylation and gene expression that are related to programmed differences in cardiomyocyte and cardiac physiology.

### Effects of developmental hypoxia on the cardiovascular response to anoxia and reoxygenation

Similar to previous in situ [45] and in vivo [44] studies on turtles, acute anoxic exposure had negative chronotropic and inotropic effects in the N21 cohort. At the end of acute anoxia, systemic and pulmonary blood flow ($\dot{Q}_{{{\text{Sys}}}}$, and $\dot{Q}_{{{\text{Pul}}}}$) in N21 turtles was significantly reduced by 51% and 44%, respectively, leading to an increase in the R–L shunt. The reduction in total blood flow ($\dot{Q}_{{{\text{Total}}}}$) in the N21 cohort was achieved by a pronounced bradycardia, while stroke volume ($V_{{{\text{S}},{\text{Total}}}}$) remained unchanged, indicating a reduction in cardiac inotropy (contractility). These results align with previous work that demonstrates vagally mediated bradycardia in anoxic turtles at 21–25 °C [44, 46] as well as negative inotropy driven by intracellular acidosis, a reduction in calcium uptake, and energy depletion [20, 47]. Reduced cardiac activity during anoxia is characteristic of turtles and serves to lower ATP demand below the capacity for anaerobic ATP supply to restore energy balance [48]. Indeed, cardiac power output remained relatively stable during anoxia, indicating that ATP supply and demand were matched [48].

In contrast to the N21 cohort, H10 turtles maintained systemic and pulmonary blood flow ($\dot{Q}_{{{\text{Sys}}}}$, and $\dot{Q}_{{{\text{Pul}}}}$,) throughout the anoxic period. Maintenance of total blood flow ($\dot{Q}_{{{\text{Total}}}}$) was supported by a blunted bradycardia and an increase in stroke volumes ($V_{{{\text{S}},{\text{Sys}}}}$ and $V_{{{\text{S}},{\text{Pul}}}}$), while ventricular pressure and cardiac power output stayed constant. Surprisingly, in contrast to previous in vivo and in situ studies of anoxia-tolerant turtles (*Trachemys scripta* and *Chrysemys picta*) [44, 49, 50], 2 h of anoxia in H10 snapping turtles led to an increase in total blood flow ($\dot{Q}_{{{\text{Total}}}}$) and maintenance of cardiac pumping capacity. These findings are remarkable when considering the 30 °C body temperature of turtles in the current study compared to prior studies of animals held at 22 °C [44, 49, 50]. The only other vertebrate known to increase total blood flow ($\dot{Q}_{{{\text{Total}}}}$) and maintain pumping capacity during anoxia is the crucian carp, *Carassius carassius*, a species that can survive anoxia for months while remaining active [51]. Nevertheless, the crucian carp study was performed at 8 °C, which would significantly reduce ATP demand. To our knowledge, cardiac performance of H10 turtles during 2 h of anoxia is unsurpassed among vertebrates.

Similar to other studies [47, 52, 53], anoxic N21 and H10 snapping turtle hearts recovered full functionality when normoxia was restored. This rare adaptation is noteworthy, because atrial reoxygenation is nearly instantaneous in turtles [54], which could theoretically expose the heart to oxidative damage through the overproduction of reactive oxygen species (ROS). Although ROS are key signalling molecules and an inevitable product of electron transport, they can also be harmful by causing lipid peroxidation, protein and DNA damage, and apoptosis [55]. In mammals, ROS production during reperfusion is a major cause of ischemia/reperfusion injury [56]. In contrast, turtles fully recover contractile function after several hours of anoxia without any conspicuous injury [47, 57, 58]. The lack of reoxygenation injury in turtle hearts has been attributed to the maintenance of an ATP/ADP pool and low succinate accumulation, which reduces the likelihood of superoxide production [59]. Interestingly, we recently found that ROS production after anoxia was significantly lower in H10 vs. N21 snapping turtle cardiomyocytes [20], which may help to explain why H10 turtles in the present study could maintain higher levels of cardiac performance during reoxygenation (Fig. 1).

### Effects of developmental hypoxia on cardiovascular regulatory mechanisms

Drawing from previous literature, we can make some inferences about the physiological and molecular mechanisms underlying differences in cardiac performance between H10 and N21 turtles. The H10 group had a blunted anoxic bradycardia compared to the N21 cohort, suggesting differences exist in factors that regulate heart rate, including local control mechanisms and/or intrinsic pacemaker properties. Acute anoxic bradycardia in warm turtles is mostly vagally mediated, but intrinsic pacemaker rate is also reduced by chronotropic extracellular factors such as acidosis, hyperkalemia, hypercalcemia, or adrenaline levels [46, 60]. Thus, developmental hypoxia could reduce the anoxia sensitivity of any of these pathways, leading to blunted anoxic bradycardia. Interestingly, H10 embryos are tachycardic when measured in normoxia and compared with N21 embryos. This is due to a blunted cholinergic tone, but the heart rate response to hypoxia was similar between groups [61]. We recently showed intracellular pH in anoxic snapping turtle cardiomyocytes is similar between N21 and H10 cohorts, but it is possible that extracellular pH, K^+^, Ca^2+^, or adrenaline levels differ, which could affect intrinsic pacemaker rate [60].

Enrichment analysis of differentially methylated genes in the current study revealed groups of genes that are functionally related to intrinsic pacemaker mechanisms, including GO terms “potassium channel complex,” “cation channel complex,” “transmembrane transporter complex,” and “ion channel complex.” We confirmed differential expression of one candidate from this category: sodium voltage-gated channel alpha subunit 5 (*SCN5A*) was up-regulated in hearts of 9-month-old turtles exposed to hypoxia as embryos. SCN5A mediates voltage-dependent sodium ion permeability of excitable membranes and inactivation of this channel is regulated by intracellular calcium. Knockout of *SCN5A* in mice results in embryonic lethality, with major defects in ventricular morphology [62]. More significantly, heterozygotes with one good copy of *SCN5A* survive, but display defects in atrioventricular and intramyocardial conduction, increased ventricular refractoriness, and tachycardia. Thus, elevated *SCN5A* expression could have the opposite effect and contribute to the higher heart rate of turtles from hypoxic incubations when exposed to acute anoxia.

Likewise, differentially methylated genes were enriched for genes that could alter autonomic control of heart rate, including GO Biological Process terms for “regulation of trans-synaptic signaling,” “regulation of nervous system development,” and “regulation of neuron differentiation,” and GO Cellular Component terms such as “synapse,” “glutamatergic synapse,” and “postsynaptic membrane.” In line with these observations, analysis of differentially methylated CGIs (i.e., potential enhancers) revealed significant enrichment of binding sites for Paired-Like Homeobox 2B (*PHOX2B*). This gene is intriguing, because it plays a key role in the formation of autonomic reflex pathways, including those controlling baroreflexes [63]. Mutation of *PHOX2B* in humans causes congenital central hypoventilation syndrome, which has cardiac arrhythmia as one of its incompletely penetrant symptoms [64]. Previous work has shown that hypoxic incubation alters α-adrenergic regulation of heart rate, β-adrenergic regulation of blood pressure, and influences expression of α- and β-adrenoreceptors in snapping turtle embryos [12]. Those findings point to hypoxic programming of the autonomic nervous system and/or tissue responsiveness to sympathoadrenal regulation. Further work is needed to identify mechanisms underlying the contribution of parasympathetic and sympathetic branches to the blunted bradycardic response in H10 turtles.

In addition to differences in the heart rate response to anoxia between experimental groups, anoxia led to an increase in cardiac inotropy in the H10 cohort, while this parameter was decreased in the N21 cohort. These results reflect the finding that N21 cardiomyocyte contractility remains depressed throughout 20 min of anoxia, while H10 contractility rebounds to pre-anoxic levels [20]. Improved anoxia tolerance of H10 cardiomyocytes was supported by enhanced myofilament calcium sensitivity and a superior ability to suppress ROS production [20]. Therefore, the increase in stroke volume observed in anoxic H10 turtles might be partly driven by intrinsic regulation of calcium and ROS. In support of this idea, we confirmed differential expression of peptidylprolyl isomerase A (PPIA) between N21 and H10 turtles. Secretion of this protein occurs in response to oxidative stress and plays a role in mediating Angiotensin II effects on cardiomyocyte hypertrophy by potentiating ROS production [65].

Enrichment analysis of differentially expressed genes in the current study identified other genes that might contribute more directly to positive inotropy in vivo and enhanced cardiomyocyte contractility in vitro: relevant GO Biological Process terms included “sarcomere organization” and “signal transduction,” as well as GO Cellular Component terms “z-disc” and “collagen trimer.” We confirmed differential expression of two candidate genes in this category: troponin T2, cardiac type (TNNT2) and tropomyosin 3 (TPM3) were both elevated in hearts of turtles exposed to hypoxia as embryos. Together, these proteins regulate calcium-dependent contraction of myofilaments. Increased TNNT2 and TPM3 expression could, therefore, cause differences in calcium sensitivity of cardiomyocytes in vitro [20] and contribute to the physiological differences observed in vivo in the present study.

### Developmental hypoxia programs genome-wide DNA methylation and gene expression patterns

While examples of individual candidate genes discussed above are interesting, our study provides the first ever genome-wide analysis of DNA methylation and gene expression patterns in a reptile. WGBS allowed us to characterize the methylation landscape across the snapping turtle genome in unprecedented detail. Promoters and first exons displayed a bimodal pattern of CpG methylation, while other genome features displayed a unimodal distribution. CpGs were more highly methylated in genes than intergenic regions, which displayed a broader range of methylation levels. We also observed genome-wide correlations between CpG methylation and gene expression in the snapping turtle that are consistent with correlations observed in mammals, anuran amphibians, and fish [66]. In particular, methylation levels in promoters and first exons were negatively correlated with mRNA expression, whereas methylation in the remaining exons and introns was positively correlated with mRNA expression. Finer scale spatial analysis of CpG methylation across proximal promoters and the 5′ end of genes revealed a clear signature: higher methylation at TSSs was associated with lower expression, while lower methylation at TSSs was associated with higher expression (Fig. 5). These findings indicate DNA methylation near promoters plays a conserved role in repression of gene expression in turtles. The discovery of a positive correlation between methylation in gene bodies and gene expression in turtles is also observed in other vertebrate lineages [66]. It has been suggested that this positive correlation is a secondary effect of the greater accessibility of more highly transcribed genes to DNA methylating enzymes [67]. Together, these observations are significant, because DNA methylation is absent (or minimal) and plays no role in regulating gene expression in some model organisms [68, 69].

We also found that exposure to hypoxic conditions during embryogenesis programmed DNA methylation patterns and that methylation of CpGs vs. CGIs varied among genomic features. Intergenic regions, where enhancers and silencers are located, were enriched for differentially methylated CGIs but not for individual CpGs. If orphan CGIs in turtles act as enhancers, as suggested by recent studies in mammals [34–36], differential methylation of these sites might play an outsized role in driving differences in gene expression patterns between N21 and H10 turtles. In contrast to intergenic regions, promoters were less likely to contain differentially methylated CGIs but were enriched for differentially methylated CpGs. These findings are consistent with the observation that CGIs in promoters are usually unmethylated in mammals [37]. Finally, we found that exons displayed less differential methylation than expected by chance for both CpGs and CGIs. Overall, we detected significant relationships between hypoxia-induced DNA methylation, gene expression patterns, and cardiovascular physiology later in life, though links for individual gene are not linear and will require substantial experimental work to elucidate.

Nonetheless, we were able to gain insight into potential regulatory mechanisms through the identification of enriched sequence motifs (i.e., putative TF-binding sites) in promoters of differentially expressed genes. For instance, glucocorticoid response elements were enriched in proximal promoters of genes that were differentially expressed between ventricles of snapping turtles from hypoxic vs. normoxic incubations. Work in mammals shows that glucocorticoids play a role in maturation of the cardiovascular system during late gestation, including effects on peripheral resistance, blood pressure, and heart rate that protect against acute hypoxia in embryos [70]. Reciprocal interactions between hypoxia and glucocorticoids have also been observed: fetal hypoxia programs differential methylation and expression of the glucocorticoid receptor gene in rat hearts [71]. Although we did not detect differential methylation or expression of the glucocorticoid receptor gene in the snapping turtle, a potential link between hypoxia-induced differential gene expression and signaling via glucocorticoid response elements deserves further study. Perhaps there are programmed differences in the function of the hypothalamic–pituitary–adrenal axis between N21 and H10 turtles, as observed in adult rats exposed to intermittent hypoxia during the postnatal period [72].

Another proximal *cis*-regulatory element might play a major role in driving differential gene expression between hearts of N21 and H10 turtles. Promoters were enriched for sequence motifs recognized by Ras Responsive Element-Binding Protein 1 (*RREB1*). Mutations in *RREB1* in humans cause Noonan-spectrum disorders, which are recapitulated in *Rreb1* hemizygous mice [73]. Mice with one functional copy of *Rreb1* exhibit cardiac hypertrophy and sensitization of cardiomyocytes to MAPK signaling. Interestingly, “signal transduction” was an over-represented GO term among differentially expressed genes, which included *MAP3K5* and *MAPK9*, in turtle hearts. Anastasiadi et al. [66] also found enrichment of *RREB1*-binding sites in genes (gene body ± 4 kb) that display tissue-specific differential methylation. The observation that *RREB1* is a methyl-CpG-binding TF suggests it may play a broader role in regulating differential gene expression in a DNA methylation-dependent manner [29].

We also identified sequence motifs that were significantly enriched within differentially methylated CGIs. We hypothesize these putative TF-binding sites are involved in the initial programming of differential DNA methylation of specific CGIs during embryogenesis as well as causing differential expression of target genes and differences in cardiovascular physiology later in life. Future studies using promoter capture HiC or HiCap could be used to test whether differentially methylated CGIs physically interact with promoters of differentially expressed genes [74, 75], as predicted by our model. However, it is not a straightforward task to link putative enhancers to their target promoters, because enhancers can act at distances of tens to hundreds of thousands of bases via DNA looping and skip over other promoters [74–76]. Those studies report that only a portion of enhancers (47% to 65%) physically interact with the nearest active TSS. Thus, the closest gene may not always be the target for putative “CGI enhancers” in the snapping turtle. This could explain why genes closest to differentially methylated CGIs were less likely rather than more likely to be differentially expressed in the snapping turtle. Another potential explanation is that the current version of the snapping genome is a scaffold level assembly. A fragmented genome could hamper our ability to physically associate differentially methylated CGIs with their closest targets.

Examination of transcription factors that bind enriched motifs in differentially methylated CGIs lends credence to our hypothesis. All three hypoxia inducible transcription factors (HIF1A, ARNT, and EPAS1) are in the list of enriched motifs and are candidates for programming differential DNA methylation in turtle hearts (Additional file 4: Table S4). Hypoxia can cause global changes in DNA methylation by regulating expression of DNA methyltransferases and ten–eleven translocation (TET) methylcytosine dioxygenases [77–79]. Yet, the hypoxia-induced methylation patterns observed here are specific rather than global and suggest targeting to *cis*-regulatory elements of genes that are differentially expressed between turtles from hypoxic vs. normoxic incubations. In fact, recent work shows that DNA methylation directly interferes with HIF binding and that cell-type specific methylation patterns determine responsiveness of HIF target genes to acute hypoxia [79]. We suggest an analogous mechanism could underlie programmed differences in DNA methylation and gene expression patterns between N21 and H10 turtles. Activation and binding of HIFs and TFs such as RREB1 and SMADs to specific sites would drive differential methylation of CpGs and CGIs in embryonic hearts. These programmed patterns would then cause differential gene expression and differences in cardiovascular phenotype later in life. Differentially methylated CGIs in turtle ventricles were enriched in binding sites for transcription factors associated with cardiovascular, mitochondrial, or autonomic defects in mammals (e.g., CUX2, GABPA, GSC, PHOX2B, SMAD4, and ZEB2).

SMAD4-binding sites were enriched within differentially methylated CGIs in turtle ventricles. Given that SMAD4 binds methylated sites [29], the intersection of DNA methylation and TGF-β signaling in the turtle heart is particularly intriguing. Indeed, embryonic hypoxia programmed differential expression of BMP10 and BMPR2 in turtle hearts. BMP10 is a key signaling molecule in the developing and mature heart in mammals [80, 81]. BMP10 is a member of the TGF-β protein family and binds to BMPR2 and ALK1 to trigger phosphorylation of SMAD2/3 or SMAD1/5/8, which form complexes with SMAD4. SMAD complexes then translocate to the nucleus to regulate expression of target genes. Signaling via SMAD4 in cardiomyocytes plays a crucial role in regulating sarcomere function, ion-channel gene expression, cardiomyocyte survival, and cardiac function in adult mice [82]. Gain of function mutations in *SMAD4* in humans cause Myhre Syndrome with cardiomyopathy [83]. Mutations of *BMPR2* in humans and mice also cause cardiovascular phenotypes that are associated with pulmonary arterial hypertension [84, 85]. Hautefort et al. [86] have recently shown that rats heterozygous for a *BMPR2* exon deletion display changes in right ventricular cardiomyocyte morphology and physiology, including smaller diameter, decreased calcium sensitivity, and decreased contractility. Thus, hypoxia-induced changes in TGF-β signaling via the BMP10/BMPR2/SMAD4 axis could direct differential DNA methylation during embryogenesis and influence subsequent cardiac physiology in N21 and H10 turtles. In fact, TGF-β-dependent activation and binding of SMADs has been shown to displace the ZNF217/CoREST/DNMT3A complex and cause demethylation of the p15^ink4b^ promoter and induce expression of the p15^ink4b^ gene [87]. There is also potential for crosstalk between TGF-β signaling and MAP kinase signaling, because SMADs and RREB1 (discussed above) have been shown to directly interact to regulate epithelial to mesenchymal transitions and induce fibrosis in myofibroblasts [88].

## Conclusion

**Fig. 8:**
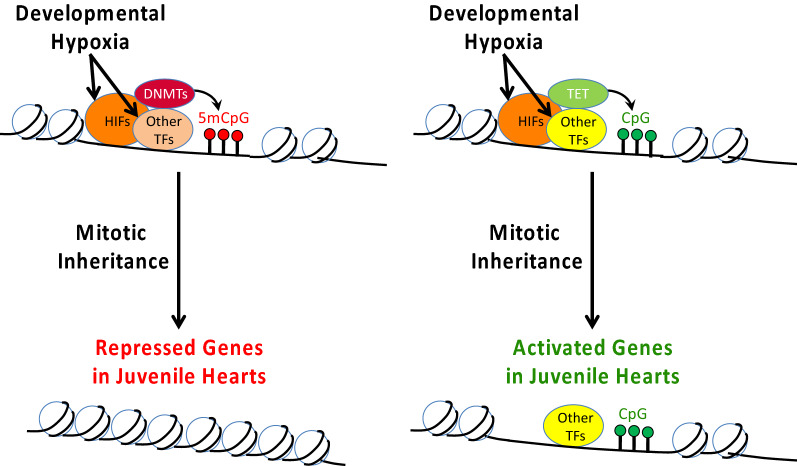
Hypothetical model for developmental programming of DNA methylation and gene patterns by hypoxic incubation. Exposure to low oxygen during embryogenesis activates HIFs and other transcription factors (TFs), which bind to specific sites in the genome of developing cardiomyocytes. These factors recruit DNMTs and TET in a locus specific manner to methylate and demethylate adjacent CpGs, respectively. DNA methylation patterns in CpGs and CGIs are inherited mitotically and influence gene expression patterns later in life

Overall, our study shows chronic hypoxia during embryogenesis significantly improves cardiac anoxia tolerance in juvenile snapping turtles and that these effects are associated with changes in DNA methylation and gene expression patterns. Our findings also point to specific genes and signaling pathways that may underlie extreme hypoxia/anoxia tolerance in the snapping turtle. Based on these findings, we propose a model in which hypoxia during embryogenesis activates hypoxia inducible factors (HIF1A, ARNT, and EPAS1) and other key TFs (e.g., RREB1 and SMAD4), which interact with specific-binding sites to direct (or inhibit) methylation of nearby CpGs and CGIs (Fig. 8). Hypoxia-induced DNA methylation patterns would then be passed down through cell divisions and maintained in later life (i.e., they are programmed). Differential methylation of CpGs and CGIs would modulate promoter/enhancer/silencer activity, chromatin structure, and influence gene expression by affecting binding of the same transcription factors (e.g., HIFs, RREB1, and SMAD4) later in life. This, in turn, would drive differences in cardiomyocyte and cardiac physiology. It is important to point out that these findings are correlative and that further research will be required to test the hypothesized mechanisms. There are limitations in the approaches that can be used in turtles. Genetic approaches such as gene knockouts and transgenics are not yet feasible with turtles. However, potential experimental approaches include primary cell culture of cardiomyocytes. An in vitro system would be amenable to pharmacological manipulations of the signaling pathways identified here. Mechanisms could also be studied by transient transfection of expression vectors with candidate genes from this study and/or knockdown of genes with siRNA or lentiviral vectors carrying shRNA.

The physiological significance of these findings also awaits further research. On one hand, increasing cardiac output during acute anoxia might be beneficial for breath-hold dives when snapping turtles are foraging. During overwintering periods, this increased capacity, combined with the low ambient water temperature, could allow the animals to sustain activity if needed. However, this strategy could become risky for longer periods of anoxia if ATP turnover is elevated and glycogen reserves become limited. In this regard, it would be interesting to measure levels of lactate production in anoxic H10 turtles to assess ATP turnover rate.

## Methods

### Turtle collection, incubation, and husbandry

Common snapping turtle (*Chelydra serpentina*) eggs were collected from the wild in Minnesota, USA, and transported to the University of North Texas, TX, USA, for incubation. Permission to collect the eggs was granted to DA Crossley by the Minnesota Department of Natural Resources (permit no. 21232). On arrival two eggs from individual clutches were staged to establish embryonic age. Eggs were embedded to their midpoint in vermiculite, inside plastic boxes (2.5-L Ziploc® containers, SC Johnson, Racine, WI, USA) that were placed inside large (75.7 L) sealable plastic bags (Ziploc®). All incubation conditions were carried out in a walk-in environmental control room (model IR-912L5; Percival Scientific, Perry, IA, USA). The vermiculate was mixed in a 1:1 ratio with water, as previously described [89]. Incubation lasted no more than 55 days and all eggs were maintained at 30 °C, a female-determining temperature [61].

At approximately 20% of development (9–12 days after laying; determined by embryonic staging), eggs were randomly assigned to either normoxic/atmospheric oxygen (21% O_2_; designated as N21) or hypoxic (10% O_2_; designated as H10) cohorts for the remainder of embryonic development. To achieve the desired oxygen level, parallel gas inflow and outflow tubes were attached to the large Ziploc® bags and O_2_ gas mixtures were set at a flow rate of 2–3 L min^−1^ using rotameters (Sho-Rate Brooks Instruments Division, Hatfield, PA, USA) downstream of either compressed N_2_/air mixture or air alone. The gas mixtures passed through an H_2_O bubbler to ensure 80–95% relative humidity and their compositions were monitored continuously with an oxygen analyser (S-3AI; AEI Technologies, Pittsburgh, PA, USA).

Upon hatching, all turtles were housed in common, normoxic (21% O_2_) conditions at 26 °C, in separate normoxic and hypoxic groups. Turtles were fed ad libitum, with dry turtle food (Mazuri, PMI Nutrition International, Brentwood, MO, USA) 2–4 times weekly and kept in a daily 12:12 light–dark cycle until experimentation (up to 1.5 years).

### In situ turtle cardiac anoxia tolerance

Size- and clutch-matched turtles from each developmental cohort were studied 1.5 years after hatching (*N* = 6 and 5, for N21 and H10, respectively). Turtle body and heart masses are provided in Table 1. Prior to experimentation, turtles were anaesthetized in a sealed box containing cotton gauze saturated in isoflurane (Isoflo®, Abbott Laboratories, North Chicago, IL, USA). Once pedal and eye reflexes were absent, turtles were removed from the box, placed ventral-side up and intubated with flexible Tygon® tubing that was inserted into the trachea via the glottis. A ventilator (model 683, Harvard Apparatus, Holliston, MA, USA) and vaporizer (FluTec vaporizer, FluTec, Ohmeda, OH, USA) provided mechanical ventilation with 3% isofluorane, at a rate of 3–4 breaths min^−1^ and tidal volume of 20 mL kg^−1^. A gas-mixer (GF-3mp, Cameron Instrument Company, Port Aransas, TX, USA) was connected to the ventilator and controlled the composition of gases.

A square cut (4 cm^2^) was made in the plastron directly over the heart to expose the major cardiac outflow vessels and pericardium. Major arteries were isolated from surrounding tissue by blunt dissection for placement of the blood-flow probes (Transonic Systems, Ithica, NY, USA). One probe (3- or 4-mm diameter) was used to measure blood flow in the right aorta, both subclavian arteries, and the right carotid collectively. Separate probes were used to measure blood flow in the left aortic, left carotid artery (both 1–2 mm), and the left pulmonary artery (1.5–2.5 mm). Each flow probe was calibrated at 30 °C, with an infusion syringe pump (PHD 2000, Harvard Apparatus, USA). The flow probes were connected to two T206 blood-flow meters (Transonic Systems Ithica, NY, USA). To measure ventricular pressure, a small hole was made in the apex of the heart using a 22-gauge needle and a pressure catheter (size 1.4 F, model SPR-671, Millar Instruments, Houston, TX, USA) was inserted into the lumen of the heart. The catheter was connected to an amplifier (MPVS-300, Millar Instruments) which was calibrated daily against a static column of water, using a two-point calibration (0 and 1 kPa). The outputs from the flowmeters and pressure amplifier were connected to a PowerLab® 8/35 data-recording system (ADInstruments, Colorado Springs, CO, USA) and recorded on a computer, with LabChart Pro® software (v8.2, ADInstruments), and data were recorded at 100 Hz.

After the flow probes and catheters were placed, isofluorane was reduced to 1–1.5%, ventilation was raised to 10–11 breaths min^−1^, and cardiovascular variables were left to stabilize for at least 30 min before the experimental protocol commenced. The experiment was designed to measure cardiac function during three distinct periods: 10 min of normoxia (21% O_2_, 3% CO_2_, and 76% N_2_), 120 min of anoxia (3% CO_2_ and 97% N_2_), and 30 min of reoxygenation (21% O_2_, 3% CO_2_, and 76% N_2_). The ventilated gas mixture was regularly checked with oxygen and carbon-dioxide analyzers (model S-3A/I and CD-3A, respectively, Ametek, Berwyn, PA, USA). All studies were carried out according to an approved animal-care protocol of the University of North Texas Institutional Animal Care and Use Committee (no. 1403-04).

Mean blood-flow (*Q̇*) values were calculated from the average of 5-min data periods throughout the experimental protocol. Total systemic blood flow (*Q̇*_Sys_) was calculated as the sum of flow from the right and left aortas, subclavian arteries, and carotid arteries, whereas total pulmonary blood flow (*Q̇*_Pul_) was calculated as 2× the flow of the left pulmonary artery, assuming that flows through the left and right pulmonary arteries are identical. Total cardiac output (*Q̇*_Tot_) was calculated as the sum of *Q̇*_Sys_ and *Q̇*_Pul_. Total, systemic, and pulmonary stroke volumes ($V_{{{\text{S}},{\text{Tot}}}}$, $V_{{{\text{S}},{\text{Sys}}}}$, and $V_{{{\text{S}},{\text{Pul}}}}$, respectively) were calculated using the following equation:

1 $$ V_{{\text{S}}} = \frac{{\dot{Q}}}{{f_{{\text{H}}} }}, $$

where *Q̇*_Tot_, *Q̇*_Sys,_ and *Q̇*_Pul_, were used to find $V_{{{\text{S}},{\text{Tot}}}}$, $V_{{{\text{S}},{\text{Sys}}}}$, and $V_{{{\text{S}},{\text{Pul}}}}$, respectively.

Net and fractional shunts were calculated using Eqs. 2 and 3, respectively, to assess the distribution of blood flow between the pulmonary and systemic circulations:

2 $$ \dot{Q}_{{{\text{Shunt}}}} = \dot{Q}_{{{\text{Pul}}}} - \dot{Q}_{{{\text{Sys}}}} , $$

3 $$ \dot{Q}_{{{\text{Fractional}}}} = \frac{{\dot{Q}_{{{\text{Pul}}}} }}{{\dot{Q}_{{{\text{Sys}}}} }}. $$

Mean ventricular pressure ($P_{{{\text{Vent}}}}$) was calculated using the following equation:

4 $$ P_{{{\text{Vent}}}} = \frac{{P_{{{\text{Systolic}} }} + 2P_{{{\text{Diastolic}}}} }}{3}, $$

where $P_{{{\text{Systolic}} }} \;{\text{and}}\;P_{{{\text{Diastolic}}}}$ are systolic and diastolic pressure, respectively.

Finally, cardiac power output (PO) was calculated using the following equation:

5 $$ {\text{PO }} = \frac{{\dot{Q}_{{{\text{Total}}}} \cdot \Delta P}}{{{\text{heart}}\;{\text{mass}}}}, $$

where $$\Delta P = P_{{{\text{Systolic}} }} - P_{{{\text{Diastolic}}}}$$.

### Transcriptome analysis of hearts exposed to developmental hypoxia

RNA-Sequencing (RNA-Seq) was carried out to measure steady state differences in cardiac gene expression between juvenile turtles exposed to normoxic or hypoxic conditions during embryogenesis. Normoxic and hypoxic groups included 7-month-old (*n* = 5) and 9-month-old turtles (*n* = 3), for a fully factorial design (total *n* = 16). The hypoxic group included equal numbers of turtles with normal-sized (*n* = 4) and enlarged hearts (*n* = 4) relative to their body size.

Hearts were dissected from turtles and weighed. Atria and ventricles were separated, placed in microfuge tubes, snap frozen in liquid nitrogen, and stored at − 80 °C. Total RNA was isolated from ventricles by grinding frozen tissue with a mortar and pestle on dry ice. Frozen, pulverized tissue was transferred to a tube containing Trizol and homogenized for another 30 s using a BioGen PRO200 homogenizer with a 5 mm generator probe. Remaining steps were carried out according to the manufacturer’s protocol. The only modification was 2 additional extractions with 500 µL of chloroform to remove phenol traces from the aqueous phase prior to RNA precipitation. RNA quality was high with RINs ranging from 8.4 to 9.1 and no indication of genomic DNA contamination when assessed on agarose gels or via qPCR (no amplification).

Total RNA was used as input for the NEB PolyA nondirectional library preparation kit. Barcoded cDNA libraries with 250–300 bp insert sizes were sequenced on Illumina HiSeq system (150 bp, paired end reads) by Novogene. One set of 6 samples was sequenced to a depth of 29 to 33 million raw reads (forward + reverse), while a second set of 10 samples was sequenced to a depth of 65 to 150 million raw reads (forward + reverse) (Additional file 5: Table S5). The first 11 bp of reads were cropped and low-quality bases trimmed (sliding window of 4 bp and average *Q* score ≥ 15) with a minimum read length of 30 bp using Trimmomatic [90]. Reads were mapped to the snapping turtle genome [91] using HISAT2 with default parameters [92]. featureCounts [93] was used to extract read counts from BAM files for subsequent gene expression analyses.

DESeq2 was used to screen for differences in gene expression [94]. Oxygen concentration, age, and the oxygen concentration by age interaction were independent factors in a general linear model with a genewise *P* < 0.01. The distribution of FPKMs were manually examined to identify differences driven by outliers. Two-way ANOVAs were then carried out on FPKMs for each gene identified by DESeq2 to ensure differences were significant using two different statistical models. DESeq2 uses a hierarchical model with likelihood ratio tests and shrinks estimates of dispersion by assuming genes with similar expression values display similar variance. In contrast, ANOVA employs ordinary least squares with *F* tests that use empirically derived variance estimates for each gene. Genes were excluded from the final list of differentially expressed genes when an outlier drove a significant effect or when DESeq2 and two-way ANOVA results were not concordant (i.e., differences were not robust to the statistical model). Gene expression was also compared between normal-sized hearts (*n* = 12) vs. enlarged hearts (*n* = 4) with an FDR adjusted *p* value < 0.1. The final set of differentially expressed genes included those affected by oxygen concentration, the oxygen concentration by age interaction, and the genes that differed between normal-sized (both N21 and H10) vs. enlarged hearts (H10). Genes that only changed with age were not analyzed any further, because the long-term effect of hypoxia was the primary focus of this study.

### Validation of differential gene expression in hearts exposed to developmental hypoxia

qPCR was used to measure gene expression in a larger set of samples from the same experiment that produced animals for the RNA-Seq study (i.e., 13 normoxic hearts and 12 hypoxic hearts; 20 normal-sized hearts and 5 enlarged hearts). Total RNA was extracted as described above. Reverse transcription and absolute qPCR with rigorous standard curves were carried out as previously described [61, 95]. Expression of *CACNA2D1*, *CNP*, and *YTHDF3* were not affected by any independent variables so these genes were used as controls. The first component from a principal components analysis of these genes was used as a covariate for analysis of the remaining genes. This covariate serves as a control for variation in the quality of input RNA and the efficiency of reverse transcription reactions (i.e., such as a housekeeping gene).

### Methylome analysis of hearts exposed to developmental hypoxia

Turtles exposed to normoxic (*n* = 3) or hypoxic conditions (*n* = 3) during embryogenesis were used for WGBS. DNA was extracted from frozen, pulverized ventricles of the same 9-month-old turtles used for the RNA-Seq study. DNA was extracted using the DNeasy Blood and Tissue kit from Qiagen. Agarose gel electrophoresis of DNA revealed high molecular weight DNA (> 60 kb) with no RNA contamination. Six μg of DNA was shipped to Novogene for WGBS. Libraries were prepared with 200–400 bp insert sizes. Bisulfite (BS) conversion was carried out with the EZ DNA Methylation Gold Kit from ZymoResearch. Libraries were sequenced on a NovaSeq 6000 instrument. QC analysis of raw reads showed BS conversion rate was greater than 99.9% for all libraries and coverage ranged from 32.9× to 37.6× (Additional file 6: Table S6).

Trimmomatic was used to remove TruSeq3 PE adapters, trim 3 bp from the 5′ and 3′ ends, and trim low-quality bases (sliding window of 4 bp and average *Q* score ≥ 15) with a minimum read length of 36 bp [90]. Reads were mapped to the snapping turtle genome using the Bismark bisulfite read mapper [96]. Mapping statistics for each library are summarized in Additional file 7: Table S7. Average mapping efficiency was 79.2%, which is excellent for WGBS data [97]. As expected, methylated cytosines were primarily found in the context of CpG dinucleotides (75%). Few methylated cytosines were found in the context of CHG (0.2%) or CHH (0.2%) trinucleotides, where H is any base except G. Approximately 3.4% of methylated cytosines were in an unknown context.

methylKit was used to call methylated CpGs and determine whether methylation levels were significantly different between N21 and H10 groups [98]. A minimum coverage of 10 in two of three replicates was required for statistical comparison. Differences between N21 and H10 groups were called significant for individual CpGs if the difference in methylation was > 25% and *q* < 0.01 (*q* is the FDR adjusted *p* value).

We used the newcpgreport tool (https://www.bioinformatics.nl/cgi-bin/emboss/newcpgreport) to call CGIs in the snapping turtle genome using default parameters: Obs/Exp > 0.6, %C + %G > 50, and length > 200 bp. We identified 201,828 CGIs in the snapping turtle genome which is less than the 307,193 CGIs in the human genome with the same parameters [37]. When corrected for genome size, however, the frequency of CGIs is similar at 89,383 CGIs/Gb in the snapping turtle and 93,089 CGIs/Gb in humans. Overall methylation of CGIs was calculated as the sum of methylated CpGs divided by the total number of CpGs within an island, which is essentially the average % methylation across the island. Comparisons between N21 and H10 groups were made using the Fisher Exact test and *q* < 0.05.

### Statistical analyses

Data were analyzed for statistical significance by a mixed-effects, generalized linear model (GLM), using Šidák post-hoc corrections for pairwise comparisons, with SPSS 25 (IBM, Armonk, NY, USA). For the GLMs, developmental oxygen (normoxia or hypoxia), acute oxygen treatment (normoxia, anoxia, or reoxygenation), and time were the independent variables and cardiovascular variables were the dependent variables. Significance was accepted when *p* ≤ 0.05. All data are reported as means ± standard error (SEM). GOATOOLS [99] was used to test for functional enrichment of gene ontology (GO) terms among differentially expressed genes from the RNA-Seq study and differentially methylated genes from the WGBS study. Genes identified in those experiments were compared to a species-specific list of GO terms generated by Das et al. [91].

## Supplementary Information

**Additional file 1: Table S1.** Genes that were differentially methylated between ventricles from 9-month-old snapping turtles that were exposed to normoxia (N21) or hypoxia (H10) during embryonic development. Genes were classified as differentially methylated when they contained ≥ 1 differentially methylated region within their promoter and/or gene body at a *q* < 0.001.

**Additional file 2: Table S2.** Gene Ontology categories and terms that were significantly enriched among 1582 genes that were differentially methylated in ventricles from 9-month-old snapping turtles exposed to normoxia (N21) or hypoxia (H10) during embryonic development. GO terms were considered significant at a Bonferroni corrected *p* ≤ 0.05.

**Additional file 3: Table S3.** Results of HOMER2 de novo motif enrichment analysis of promoters from 443 genes that were affected by oxygen concentration during embryogenesis (Table 2), the oxygen concentration by age interaction (Table 3), and/or those genes that differed between ventricles from turtles that had normal-sized vs. enlarged hearts relative to their body size (Table 4).

**Additional file 4: Table S4.** Results of HOMER2 motif enrichment analysis of 6666 CpG islands that were differentially methylated in ventricles from 9-month-old snapping turtles exposed to normoxia (N21) or hypoxia (H10) during embryonic development. The 6666 CpG islands were compared to 1,065,536 background sequences from the snapping turtle genome.

**Additional file 5: Table S5.** Summary of RNA-Seq data from ventricles of juvenile snapping turtles exposed to normoxia (N21) or hypoxia (H10) during embryonic development and sampled at 7 months or 9 months of age.

**Additional file 6: Table S6.** Summary of WGBS data from ventricles of juvenile snapping turtles exposed to normoxia (N21) or hypoxia (H10) during embryonic development and sampled at 9 months of age.

**Additional file 7: Table S7.** Summary of mapping statistics for WGBS libraries from ventricles of juvenile snapping turtles exposed to normoxia (N21) or hypoxia (H10) during embryonic development and sampled at 9 months of age.

## Data Availability

The datasets during and/or analysed during the current study available from the corresponding author on reasonable request.
